# Crystal structure and functional properties of the human CCR4-CAF1 deadenylase complex

**DOI:** 10.1093/nar/gkab414

**Published:** 2021-05-26

**Authors:** Ying Chen, Elena Khazina, Elisa Izaurralde, Oliver Weichenrieder

**Affiliations:** Department of Biochemistry, Max Planck Institute for Developmental Biology, Max-Planck-Ring 5, D-72076 Tübingen, Germany; Department of Biochemistry, Max Planck Institute for Developmental Biology, Max-Planck-Ring 5, D-72076 Tübingen, Germany; Department of Biochemistry, Max Planck Institute for Developmental Biology, Max-Planck-Ring 5, D-72076 Tübingen, Germany; Department of Biochemistry, Max Planck Institute for Developmental Biology, Max-Planck-Ring 5, D-72076 Tübingen, Germany

## Abstract

The CCR4 and CAF1 deadenylases physically interact to form the CCR4-CAF1 complex and function as the catalytic core of the larger CCR4-NOT complex. Together, they are responsible for the eventual removal of the 3′-poly(A) tail from essentially all cellular mRNAs and consequently play a central role in the posttranscriptional regulation of gene expression. The individual properties of CCR4 and CAF1, however, and their respective contributions in different organisms and cellular environments are incompletely understood. Here, we determined the crystal structure of a human CCR4-CAF1 complex and characterized its enzymatic and substrate recognition properties. The structure reveals specific molecular details affecting RNA binding and hydrolysis, and confirms the CCR4 nuclease domain to be tethered flexibly with a considerable distance between both enzyme active sites. CCR4 and CAF1 sense nucleotide identity on both sides of the 3′-terminal phosphate, efficiently differentiating between single and consecutive non-A residues. In comparison to CCR4, CAF1 emerges as a surprisingly tunable enzyme, highly sensitive to pH, magnesium and zinc ions, and possibly allowing distinct reaction geometries. Our results support a picture of CAF1 as a primordial deadenylase, which gets assisted by CCR4 for better efficiency and by the assembled NOT proteins for selective mRNA targeting and regulation.

## INTRODUCTION

The 3′-poly(A) tail is a defining feature of eukaryotic mRNAs, and the control over its presence and length plays a crucial role in the post-transcriptional regulation of gene expression (1–3). The CCR4-NOT complex (4–6) removes poly(A) tails down to the body of the mRNA (7–10), and it serves as a central regulatory node for 3′-deadenylation, for the repression of mRNA translation (11–13), and for the control of 5′–3′ mRNA decay (14–16). Several regulatory pathways converge at the CCR4-NOT complex, resulting in the recruitment of the complex to the respective mRNA targets. These pathways include the miRNA-mediated mRNA silencing pathway, pathways of protein-mediated mRNA regulation and decay that depend on specialized RNA-binding proteins, and mRNA quality control pathways, such as nonsense mediated mRNA decay (15,17,18).

The length of the poly(A) tail is also controlled by other deadenylases in the cell, distinct from the CCR4-NOT complex. PARN was identified early on as a highly specific poly(A) 3′-exoribonuclease and operates as a homodimer (19,20). PARN is however not generally conserved in eukaryotic species and does not function in general mRNA turnover (8,10). Rather, it is important in specialized cellular processes (21) and for the maturation of nuclear non-coding RNAs (22,23). The PAN2 nuclease, in contrast, plays a widely conserved role in general mRNA deadenylation. A single copy of PAN2 associates with an asymmetric dimer of the PAN3 protein, which serves as a scaffold to form the PAN2-PAN3 complex (24,25). This complex is particularly suited to act on longer poly(A) tails that are covered by several copies of the cytoplasmic poly(A) binding protein (PABPC1) as recently also demonstrated by structural analysis (8,10,26).

**Figure 1.**
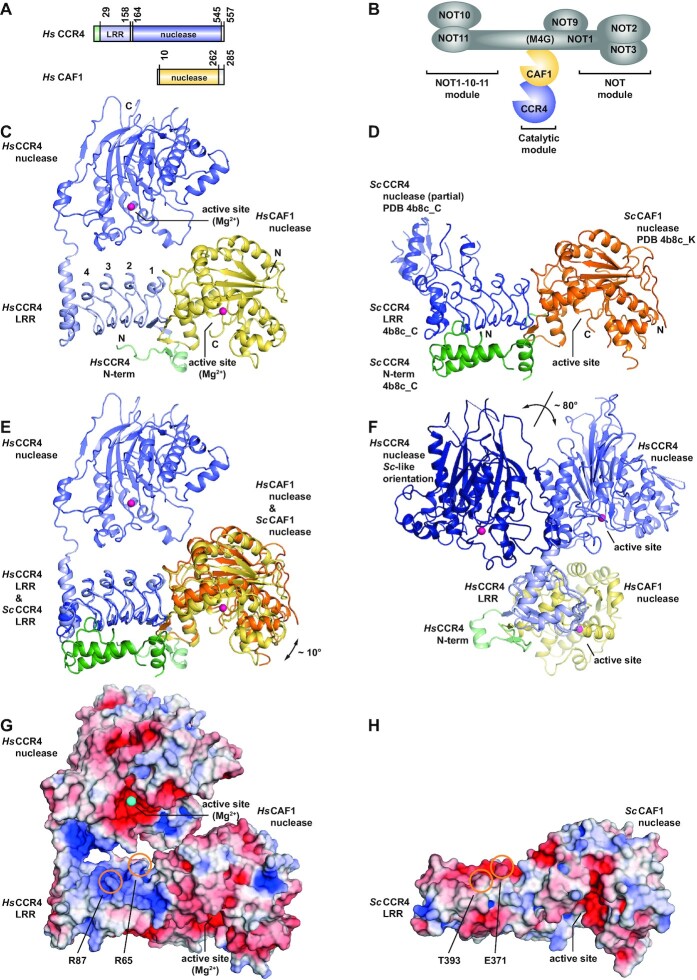
Crystal structure of the human CCR4-CAF1 mRNA 3′-deadenylation complex. (**A**) Domain composition of the human CCR4 and CAF1 deadenylases (*Hs* CCR4 and *Hs* CAF1). *Hs* CCR4 is a multi-domain EEP-type nuclease (slate) with an N-terminal extension (pale green) that is followed by an LRR domain (light blue) with four and a half leucine-rich repeats. *Hs* CAF1 is a DEDDh-type single-domain nuclease (yellow) with a short C-terminal tail. (**B**) Composition of the human CCR4-NOT complex and localization of the catalytic module. The NOT1 scaffold protein contains N-terminal α-helical domains that interact with NOT10 and NOT11 to form the NOT1-10-11 module, an α-helical bundle that interacts with NOT9, an α-helical connector domain and an α-helical NOT1 superfamily homology domain that forms the NOT module together with NOT2 and NOT3. The complex of the two deadenylases, CAF1 and CCR4, docks via CAF1 to the central α-helical ‘MIF4G’ domain of NOT1 (M4G) to form the catalytic module of the CCR4-NOT complex. (**C**) Crystal structure of the human CCR4-CAF1 complex (cartoon representation). N- and C-termini are marked and individual LRR repeats are numbered. Magnesium ions are shown as spheres (magenta) and indicate the position of the nuclease active sites. (**D**) Crystal structure of the yeast (*S. cerevisiae*, *Sc*) CCR4-CAF1 complex (PDB-ID 4b8c) (36) with the CCR4 LRR domain (blue) oriented as for the human complex (C). Note the unrelated structure of the N-terminal extension (green) and the distinct orientation of the CCR4 nuclease domain (dark blue). In contrast to the *Sc* CAF1 nuclease (orange), the *Sc* CCR4 nuclease is only partially resolved. (**E**) Superposition of the human and the yeast CCR4-CAF1 complex via the CCR4 LRR domain. Note the ∼10° tilt of the *Sc* CAF1 nuclease as compared to the *Hs* CAF1 nuclease. The *Sc* CCR4 nuclease domain is omitted for clarity. (**F**) Alternate view of the human CCR4-CAF1 complex down the CCR4 LRR domain. The *Hs* CCR4 nuclease domain is also shown in the orientation of the *Sc* CCR4 nuclease domain present in crystals of *Sc* CCR4-CAF1 complex (dark blue, rotated by ∼80°). The observed orientations of the CCR4 nuclease domains towards the remainder of the respective complex are likely guided by crystal packing forces. (**G, H**) Electrostatic potential plotted onto the molecular surface of the human (G) and yeast (H) CCR4-CAF1 complexes. The surface of the *Hs* CCR4 LRR domain harbors a basic patch that is missing from the yeast homolog. Arginines mutated in the course of this work are indicated together with the corresponding residues on the *Sc* CCR4 LRR domain. The potential is contoured from –5 kT/e (red, acidic) to +5 kT/e (blue, basic) and the *Sc* CCR4 nuclease domain is omitted for clarity.

General mRNA deadenylation in human cells is dominated, however, by the CCR4 and CAF1 nucleases of the CCR4-NOT complex (Figure 1A, B). The two nucleases directly interact with each other to form the catalytic core of the CCR4-NOT complex (27,28), and they are responsible for the deadenylation and eventual turnover of probably almost all of the cytoplasmic mRNA molecules in a given cell (10). CCR4 (29) and CAF1 (30,31) are structurally distinct nucleases. CCR4 belongs to the heterogeneous EEP (exonuclease-endonuclease-phosphatase) family of phosphoesterases (32–34), whereas CAF1 belongs to the DEDDh family of nucleases, which also comprises PARN and PAN2 (34,35). A crystal structure containing the CCR4 and CAF1 homologs from *Saccharomyces cerevisiae* (*S. cerevisiae*, *Sc*) (36) demonstrated how the two proteins interact via a flexibly linked LRR domain in CCR4 that is built from leucine-rich repeats (LRRs), and that is present also in CCR4 homologs from other fungi and from metazoan species but apparently not from plants (28,37,38).

The remaining six subunits of the human CCR4-NOT complex (5) are hereafter called the NOT proteins (39,40) and have structural and/or regulatory function in cytoplasmic mRNA deadenylation, but also in other cellular processes (18,41). The NOT proteins assemble along NOT1, which serves as a central scaffold. NOT10 and NOT11 attach to the N-terminal portion of NOT1 (13,42), whereas NOT2 and NOT3 co-assemble on its C-terminal portion (43,44). Furthermore, NOT9 (a.k.a. CAF40) binds to a central domain of NOT1 right next to its MIF4G domain (45,46) and, in metazoans, also helps to recruit the NOT4 ubiquitin ligase as a facultative cofactor of the CCR4-NOT complex (47). The NOT proteins get attached to the two catalytic nucleases via the NOT1 MIF4G domain, which docks to the CAF1 nuclease in an oriented manner (36,48). Consequently, CAF1 serves as a bridge between the NOT proteins and CCR4. This structural role of CAF1 explains why CCR4 fails to assemble with the NOT proteins in the absence of CAF1 (27,49), and why the overexpression of catalytically inactivated CAF1 impairs mRNA deadenylation more strongly than catalytically inactivated CCR4 (50,51).

Multicellular organisms must regulate mRNA levels at different developmental stages and in highly differentiated cells. Some components of the CCR4-NOT complex consequently vary among protists, fungi, plants and animals (18,52–54). Furthermore, the possible benefits arising from the presence and physical association of two seemingly redundant but structurally distinct nucleases are only beginning to be understood, as for example with respect to PABPC1 and its presence on the mRNA poly(A)-tail (9, 10). Finally, many multicellular organisms encode one or even several paralogs of the CCR4 and CAF1 nucleases (53–55). In humans and other vertebrates, these are CCR4a and CCR4b (a.k.a. NOT6 and NOT6L) as well as CAF1a and CAF1b (a.k.a. NOT7 and NOT8) (8). Examples of paralog-specific functions are the requirement of mouse CAF1a in spermatogenesis (56–58), the requirement of mouse CAF1b in embryo development (49) and the role of mouse CCR4b in oocyte maturation (59) or in maintaining systemic metabolic homeostasis (60).

To understand human mRNA regulation, comparative insight into CCR4-CAF1 structure and function from distantly related species needs to be complemented by a thorough and direct analysis of the human homologs. We consequently determined a crystal structure of the human CCR4-CAF1 complex using the CCR4a and CAF1a paralogs and systematically characterized their enzymatic properties and substrate specificity in vitro and in a range of experimental conditions. The structure reveals the relative arrangement of the two nuclease domains as well as local molecular details that are relevant for substrate binding and catalysis. Furthermore, deadenylation experiments demonstrated that CCR4 and CAF1 are differentially affected by pH and that they distinguish nucleotide identity on both sides of the scissile phosphoester bond. The experiments also showed how CCR4 and CAF1 deal in an enzyme-specific manner with terminal (61–63) or interspersed (64–66) non-A nucleotides that can be part of individual poly(A) tails (67). Their properties allow the two enzymes to be both fast and precise and to stop nucleotide hydrolysis at the RNA body, where consecutive non-A nucleotides start to accumulate. Finally, CAF1 emerged as a highly tunable enzyme that is sharply responsive to the ion concentration of surrounding Mg^2+^ and Zn^2+^ and apparently is able to catalyze deadenylation in a different way upon complex formation with CCR4.

Our results support a picture of the CCR4-NOT complex with a tunable CAF1 as the central and primordial deadenylase. CAF1 gets assisted by the associated CCR4 protein to increase the efficiency of the deadenylation process and by the assembled NOT proteins for the purpose of mRNA targeting and regulation. From the perspective of a ‘CAF1-CCR4’ complex, CCR4 extends the environmental range for catalysis and helps CAF1 to deal better with obstacles such as PABPC1 or interspersed guanine nucleotides, whereas the NOT proteins mediate the programmed recruitment and accelerated deadenylation of selected mRNA targets. The present analysis provides a solid basis and reference for future analyses of the CAF1 and CCR4 deadenylases, as well as of their regulation in the context of the CCR4-NOT complex and in different cellular environments.

## MATERIALS AND METHODS

### DNA plasmid constructs

For bacterial (co-)expression of the human (*Homo sapiens*, *H. sapiens*, *Hs*) CCR4a and CAF1a proteins (*Hs* CCR4a, Uniprot Q9ULM6, and *Hs* CAF1a, Uniprot Q9UIV1) (68) in *Escherichia coli* (*E. coli*), the corresponding cDNA sequences were inserted between the XhoI and BamHI restriction sites of the plasmids pnEA-NpM (for *Hs* CCR4a) and pnYC-NpM (for *Hs* CAF1a). These plasmid backbones are derived from the pET-MCN series and produce fusion proteins carrying N-terminal MBP (maltose binding protein) tags, cleavable by the human rhinovirus 3C (HRV3C) protease (69). The respective variants of *Hs* CCR4a and *Hs* CAF1a were generated by site-directed mutagenesis with appropriate DNA oligonucleotides and confirmed by sequencing. All of the plasmid constructs generated in this study are listed in Table S1. To facilitate cross-species comparisons, we used common protein family names for the CCR4-NOT complex: NOT1, NOT2, NOT3, NOT9, NOT10, NOT11 for the NOT proteins and CCR4a, CCR4b, CAF1a, CAF1b for the catalytic subunits. Their corresponding names according to the Human Genome Organization (HUGO names) are: CNOT1, CNOT2, CNOT3, CNOT9, CNOT10, CNOT11 for the NOT proteins and CNOT6, CNOT6L, CNOT7, CNOT8 for the catalytic subunits (14,18).

### Protein expression and purification

To purify the human CCR4a-CAF1a complex, MBP-tagged CCR4 protein was co-expressed overnight with MBP-tagged CAF1 protein in *E. coli* BL21 (DE3) Star cells (Invitrogen) in LB medium at 20°C (see Tables S1 and S2 for plasmid constructs and combinations). Cells were harvested by centrifugation and pellets were stored at –80°C. On day one of protein purification, the cells were thawed and lysed using a cooled Emulsiflex-C3 (Avestin) homogenizer and cold (4°C) lysis buffer containing 50 mM potassium phosphate (pH = 7.5), 300 mM NaCl, 2 mM DTT, DNase I (5 μg/ml), lysozyme (1 μg/ml) and ‘Complete’ EDTA-free protease inhibitors (Roche). The lysate was cleared by ultracentrifugation (200 000 g, 1 h, 4°C) and the complex was immobilized and isolated from the supernatant on amylose resin (New England Biolabs), followed by elution with 25 mM d-(+)-maltose at 4°C in a buffer containing 50 mM potassium phosphate (pH = 7.5), 300 mM NaCl and 2 mM DTT. Subsequently, the MBP tags were cleaved with HRV3C protease at 4°C overnight. On day two, the samples were concentrated, and the CCR4-CAF1 complex was separated from the cleaved MBP, the HRV3C protease and excessive CAF1 protein by size-exclusion chromatography (HiLoad Superdex 200 26/60, GE Healthcare) at 18°C in a buffer containing 10 mM HEPES (pH = 7.5), 300 mM NaCl and 2 mM DTT. Immediately thereafter, the complex was concentrated again and further polished by anion-exchange chromatography (Source 15Q 4.6/100 PE, GE Healthcare), using a starting buffer with 10 mM HEPES (pH = 7.5), 75 mM NaCl and 2 mM DTT at 18°C and applying a linear gradient of 50 ml and up to 200 mM NaCl. The CCR4 and CAF1 proteins co-eluted as a sharp peak in the middle of the gradient, and the purest fractions were pooled and concentrated to 5–10 mg/ml. The anion exchange chromatography step efficiently removed remaining contaminants and remaining traces of excessive CAF1 protein. The concentrated samples were aliquoted, flash-frozen in liquid nitrogen and stored at -80°C until further use.

Using this protocol, all of the mutant CCR4-CAF1 complexes essentially behaved the same, apart from the CCR4-CAF1 (D491N/+) variant. This variant gave a lower yield and showed an additional peak in the elution profile of the anion exchange column at a higher salt concentration, but, in deadenylation assays, samples from both peaks showed the same result (poor CAF1 activity at pH < 7.0). Furthermore, to avoid cross-contamination between individual CCR4-CAF1 mutants, chromatography columns were treated with a denaturing solution of 0.1 M NaOH after each preparation.

The isolated human CCR4 nuclease domain (*Hs* CCR4a_nuc, Table S1) was expressed and purified similarly to the CCR4-CAF1 complex, but using Superdex 75 instead of Superdex 200 resin (HiLoad Superdex 75, 16/60, GE Healthcare) and 200 mM NaCl instead of 300 mM NaCl in gel filtration chromatography. Furthermore, the starting buffer for the anion exchange chromatography step contained 100 mM NaCl instead of 75 mM NaCl, causing only contaminants to bind to the column, whereas the CCR4 nuclease domain was collected in the flow-through fractions before the onset of the gradient.

The plasmid construct (pET-MCN derived pnEA-NpG backbone) for the expression of the isolated human CAF1 nuclease with an N-terminal, HRV3C-cleavable GST (glutathione-S-transferase) tag was described previously (48). The protein was expressed in *E. coli* Rosetta II (DE3) cells (Novagen) in LB medium at 17°C overnight. Purification was done similarly to the isolated CCR4 nuclease domain, but glutathione agarose resin (Machery & Nagel) was used instead of amylose resin in the affinity chromatography step, and the anion exchange chromatography step was omitted.

### Crystallization and data collection

Initial crystallization screens were carried out by vapor diffusion in sitting drops at 22°C and by mixing 200 nl sample solution (∼3 mg/ml CCR4-CAF1 in 20 mM HEPES [pH = 7.5], ∼180 mM NaCl and 2 mM DTT) with 200 nl reservoir solution. Crystalline material appeared in several conditions and was used for micro-seeding experiments, where seed solution was added to hanging drop vapor diffusion experiments at 18°C. A limited number of crystals was obtained in this way, the best of which came from an experiment where 2 μl of sample solution had been mixed with 2 μl of a 500 μl reservoir containing 100 mM MES (pH = 6.0), 100 mM MgCl_2_ and 8% PEG 6000. The crystal was cryo-protected in reservoir solution supplemented with 20% glycerol and flash-frozen in liquid nitrogen. X-ray diffraction data (Table 1) were collected at a wavelength of 0.9786 Å on a PILATUS 6M detector (Dectris) at the Proxima 1 beamline of the SOLEIL synchrotron radiation facility (Gif-sur-Yvette). They were processed and scaled in space group C2 using XDS and XSCALE (70). We decided to include data up to a resolution of 3.3 Å, where CC_1/2_ (71) dropped below 30%.

**Table 1.** X-ray data collection and refinement statistics

Data collection*	
Space group	*C*2
Unit cell	
Dimensions *a*, *b*, *c* (Å)	149.9, 63.3, 113.1
Angles *α*, *β*, *γ* (°)	90.0, 106.8, 90.0
Wavelength (Å)	0.9786
Resolution range (Å)	71.7–3.30 (3.39–3.30)
*R*_sym_ (%)	6.6 (150.5)
Completeness (%)	99.6 (99.6)
Mean *I* / σ(*I)*	11.8 (0.8)
CC $\frac{1}{2}$ (%)	99.9 (29.0)
Unique reflections	15515 (1174)
Multiplicity	3.4 (3.4)
Refinement	
*R*_work_ (%)	24.8
*R*_free_ (%)	27.6
Number of atoms	
All atoms	6233
Protein	6231
Ligands	2
Average B factor (Å^2^)	
All atoms	149.1
Protein	149.1
Ligands	139.5
Ramachandran plot	
Favored regions, %	94.4
Disallowed regions, %	0.0
RMSD from ideal geometry	
Bond lengths (Å)	0.003
Bond angles (°)	0.502

*Values in parentheses are for highest-resolution shell.

### Structure solution and refinement

The structure of the human CCR4a-CAF1a complex was solved by molecular replacement using PHASER (72) from within the CCP4 package (73). In the search for possible solutions, we used a hybrid model for the complex between CAF1 and the CCR4 LRR domain as well as a separate model for the CCR4 nuclease domain. The hybrid model consisted of human CAF1a (PDB-ID 4gmj) (48) and the CCR4 LRR domain from *S. cerevisiae* and was generated by superimposing the human structure onto the structure of the CCR4-CAF1 complex of *S. cerevisiae* (PDB-ID 4b8c) (36). For the CCR4 nuclease domain, we used the structure of human CCR4b (PDB-ID 3ngn) (29).

Initial phases were improved by several rounds of manual model building and rebuilding, each consisting of iterative cycles of model building in COOT (74) and refinement using PHENIX (75). To avoid model bias, a simulated annealing composite omit map was calculated in PHENIX and used to guide the final building cycles. In cases of missing electron density, amino acid side chains were modeled geometrically, starting from the most common rotamer, and single Mg^2+^ ions were placed to account for the difference electron density in the active sites of CCR4 and CAF1. Final refinement resulted in an *R*_work_ of 24.8% and an *R*_free_ of 27.6% (Table 1) and illustrations were prepared using PyMOL (76) with the APBS plugin (77).

### Deadenylation assays

RNA 3′-deadenylation time course experiments were routinely performed under standard assay conditions in a volume of 50 μl and using a buffer containing 50 mM HEPES (pH = 7.5), 150 mM NaCl, 2 mM MgCl_2_ and 1 mM DTT, unless indicated otherwise. Synthetic oligonucleotide substrates (Table S3) were ordered to be HPLC-purified and to contain either a 5′- or a 3′-phospholinked 6-FAM fluorescent label (carboxyfluorescein, biomers.net). For each time point separately, RNA substrates were diluted on ice and incubated with equimolar amounts of purified proteins at final concentrations of 0.6 μM for the indicated amount of time at 37°C. The reaction was stopped by adding 150 μl of formamide-containing loading dye (95% formamide, 0.05% SDS, 0.01% bromophenol blue, 17.5 mM EDTA). The reaction products were resolved on a denaturing (5.4 M urea) polyacrylamide gel (22%, 19× acrylamide : 1 x bisacrylamide), loading 1% from each sample per lane of the gel together with 10 μl loading dye and running the 3 nucleotide product marker for a distance of 6.5 cm before scanning the gel on a Typhoon Imager (GE Healthcare). Experiments were reproduced in different combinations, including multiple independent protein preparations of the CCR4 and CAF1 active site variants.

## RESULTS

### Crystal structure of the human CCR4-CAF1 complex

To characterize the catalytic core of the human CCR4-NOT complex, we coexpressed human CCR4a and CAF1a in *E. coli*, purified the complex (in the following named CCR4-CAF1) and determined a crystal structure at 3.3 Å resolution (Figure 1C and Table 1). All of the three protein domains, the CCR4 LRR domain (S29–S158), the CCR4 nuclease domain (I164–L545), and the CAF1 nuclease (Q10–L262), were clearly defined in the electron density, with the exception of some loops and disordered regions, which are not included in the model. These are CCR4 residues M1–K5, I339–G349, L399–F402, H452–T458 and P546–R557 as well as CAF1 residues M1–S9 and S264–S285 (Supplementary Figure S1). Consequently, the present model of the human CCR4-CAF1 complex is more complete than a previously determined structure of the homologs from *S. cerevisiae* (36), which lacks approximately 70% of the CCR4 nuclease domain (Figure 1D). Moreover, the proteins from *S. cerevisiae* have rather low sequence identity to human CCR4 (36%) and CAF1 (41%), where even the active site residues are altered (SEDQt instead of DEDDh, Supplementary Figure S1).

Human CCR4 only contains a short N-terminal extension (M1–K28), which is followed by four and a half LRR repeats (LRR1 to LRR5) and two C-terminal α-helices that shield the hydrophobic residues of the last, incomplete repeat unit (LRR5; Figure 1C and Supplementary Figures S1A and S2A). This N-terminal LRR domain of CCR4 binds the CAF1 nuclease in an oriented manner, whereas the C-terminal nuclease domain of CCR4 is connected to the LRR domain by a short linker (G159–R163) and fixed by crystal packing forces, apparently without specific contacts to the remainder of the complex. The linker is traceable in the electron density and assures that the CCR4 nuclease domain remains closely tethered, but nevertheless adaptable with respect to the CCR4 LRR domain and the CAF1 nuclease (Figure 1C).

### The interface of CCR4 with CAF1 in the human complex as compared to fungal species

The orientation of the human CAF1 nuclease with respect to the CCR4 LRR domain is similar to the orientation observed for the homolog from *S. cerevisiae* (36), although tilted by approximately 10 degrees (Figure 1E and Supplementary Figure S2A, B). In the interface, the hydrophobic surface of the first LRR repeat gets covered by hydrophobic residues from CAF1 α-helix α2 (C67 and L71) and by M107, as observed for the corresponding residues A215, F219 and I256 in *S. cerevisiae* (Supplementary Figure S2C, D). Furthermore, and similar to the yeast homolog as well, residues A48–P50 from CAF1 (β-strand β_L1_) engage in a short, antiparallel β-strand interaction with β-strand β1 from the first LRR repeat (Supplementary Figures S1 and S2A–D).

Other portions of the interface, however, clearly differ between the two structures and might also more generally distinguish fungal from metazoan complexes. These are the N-terminal extension of the human CCR4 LRR domain which makes additional contacts with CAF1 (Figure 1C, D and Supplementary Figures S1 and S2A, B), the metazoan-specific distortion of the first LRR repeat in human CCR4 (Supplementary Figure S2C, D) which lacks the asparagine from the typical 23 amino acid consensus (LxxLxLxxNxLxxLpxxLxxLxx; Supplementary Figures S1A and S2E–G) (78), and the conformation of the widely conserved 'GVV’ sequence (G45-V46-V47) in human CAF1 which contacts the CCR4 LRR1 repeat (Supplementary Figures S1B and S2C, D). Nevertheless, catalytically inactivated mouse CAF1 can phenotypically compensate the lack of the homolog in *S. cerevisiae* (79).

### Flexible orientation of the CCR4 nuclease domain

The orientation of the human CCR4 nuclease domain with respect to the CCR4 LRR domain and consequently also with respect to the CAF1 nuclease differs considerably from the orientation observed in the crystal structure of the homolog from *S. cerevisiae* (36), where the CCR4 nuclease domain makes contact with the LRR domain but is rotated away from the CAF1 nuclease by approximately 80 degrees (Figure 1F). The backbone of the human CCR4 nuclease domain approaches the backbones of the CCR4 LRR domain and of the CAF1 nuclease to less than 10 Å, but without a defined contact. The two active sites remain almost 50 Å apart and in an orientation that precludes them to work simultaneously on the same RNA 3′-end. A switch of the RNA 3′-end between active sites therefore requires a considerable reorientation of the terminal RNA nucleotides, despite the apparently flexible attachment of the CCR4 nuclease domain.

It is noteworthy that the convex surface of the CCR4 LRR domain, which faces the CCR4 nuclease active site in the human structure, shows an extended patch of positively charged residues (Figure 1G), including K39, R41, R65, K85, R87 and R110 (Supplementary Figures S1A and S2G, H). This patch could help to guide the negatively charged phosphoribose backbone of an RNA substrate and is present in the aligned metazoan and fungal species, with the exception of CCR4a paralogs in mouse and frog, and of the homolog from *S. cerevisiae* (Figure 1H and Supplementary Figure S1A).

### Conformational plasticity in CCR4 and CAF1 and comparison to paralogs

Regarding the CCR4 nuclease, only the nuclease domain of the CCR4b paralog had been crystallized before (29). Superposition with the CCR4a nuclease domain reveals two major conformational changes affecting two adjacent loops, the large loop L(β11–β12) and the ‘HWDP’ loop in the active site (Supplementary Figure S3A). The sequences of these two loops in CCR4a (V300–I326 and H363–P366, respectively) are identical to the CCR4b paralog, apart from the peripheral L305 (Supplementary Figure S1A). This suggests both loops to be malleable in both CCR4 paralogs. Quite generally, the systematic differences between the paralogs primarily localize to the peripheral or disordered regions of the complex rather than to the active sites (Supplementary Figures S1 and S3B).

Regarding the CAF1 nuclease, a comparable structure of the CAF1b paralog is still missing. Superposition with a previously determined structure of human CAF1a from its complex with NOT1 (48), however, also reveals two malleable regions, located near the entrance to the CAF1 active site. These are a part of the conserved loop L(β2–α2) (A48–Y60) and the C-terminal tail (G263–S285), which differs in sequence between the two paralogs (Supplementary Figures S1B and S3A). Importantly, the loop L(β2–α2) rearranges upon complex formation with the CCR4 LRR domain and folds into an additional turn of α-helix α2 and into the two β-strands β_L1_ and β_L2_, which pair with distinct β-strands from the CCR4 LRR domain (Supplementary Figures S1B, S2A, S3A).

### Active sites and RNA substrate recognition by CCR4 and CAF1

**Figure 2.**
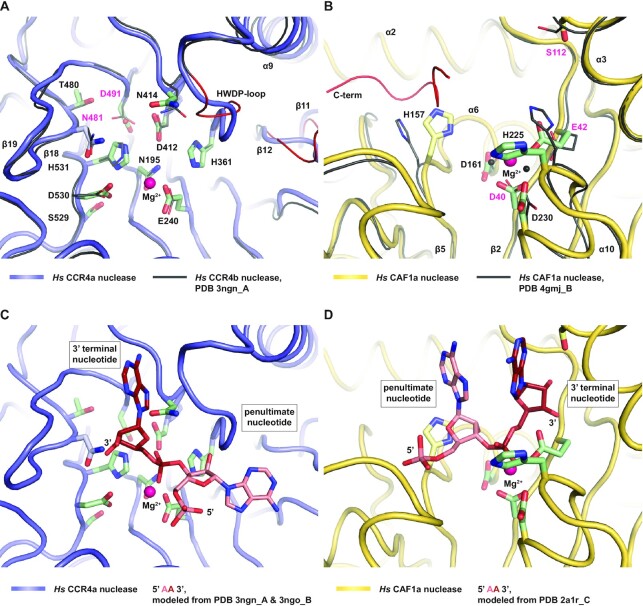
Geometry and flexibility of catalytic residues in the active sites of CCR4 and CAF1. (**A**) Active site of *Hs* CCR4a with the conserved side chains of the EEP nuclease family shown as green sticks. Other important side chains are also shown as sticks. Residues mutated in the course of this work are labeled in magenta. Superposition with the active site of *Hs* CCR4b (black, PDB-ID 3ngn) (29) demonstrates the conservation of sequence and structure apart from the HWDP loop and the loop L(β11–β12), which appear to be flexible elements (red). (**B**) Active site of *Hs* CAF1a with the conserved side chains of the DEDDh nuclease family shown as green sticks. Other important side chains are also shown as sticks. Residues mutated in the course of this work are labeled in magenta. Superposition with a previously determined structure of *Hs* CAF1a (black, PDB-ID 4gmj) (48) indicates malleability of H157 and H225 and shows the position of the C-terminal peptide (red), which may interfere with the access of an RNA substrate. (**C**) Active site of *Hs* CCR4a including a crude model of an RNA 3′-terminal end. The 3′-terminal adenine corresponds to the AMP molecule found in the structure of *Hs* CCR4b (PDB-ID 3ngn) (29). Modelling of the penultimate adenine was guided by a DNA ligand of *Hs* CCR4b (PDB-ID 3ngo) (29), followed by energy minimization. (**D**) Active site of *Hs* CAF1a including a crude model of an RNA 3′-terminal end. The two 3′-terminal adenines were placed according to a structure of the PARN 3′-deadenylase in complex with RNA (PDB-ID 2a1r) (20). Oxygen atoms, red; nitrogen atoms, blue; magnesium ions, magenta.

Amino acid side chains in the active sites of CCR4 and CAF1 were defined well enough in the electron density to determine their rotameric state (Supplementary Figure S3C, D) and were generally oriented as previously observed in the individually crystallized nucleases (Figure 2A, B). Exception were H157 and H225 in CAF1, which were modeled geometrically. They probably can adopt alternative conformations such as previously observed for H225 (48).

Difference electron density was observed in the active sites of both CCR4 and CAF1 and was interpreted by placing Mg^2+^ ions. In the case of CCR4, spherical difference electron density justified the placement of a single Mg^2+^ ion (Supplementary Figure S3C). A one-ion reaction mechanism was originally proposed for the EEP enzyme APE1, including a backbone flip of the nucleotide downstream of the scissile bond (80). This mechanism has subsequently been confirmed for APE1 by a series of crystallized intermediates (81). Nevertheless, EEP enzymes including CCR4 also get modeled with two bound metal ions (29) despite the lack of intermediate structures supporting a corresponding mechanism. In contrast, in the case of CAF1 and the DEDDh family of enzymes, a two-ion reaction mechanism (82) with stacked nucleotides around the scissile bond is well established (20,48). However, due to the limited resolution of our crystal structure, we roughly positioned only a single Mg^2+^ ion to interpret the elongated difference electron density in CAF1 (Supplementary Figure S3D).

To visualize the likely recognition modes of the penultimate and terminal nucleotides at RNA 3′-ends in the active sites of CCR4 and CAF1, and in order to guide our functional analysis, we generated structural models (Figure 2C, D). We took the structures of human CCR4b in complex with AMP (29) and of human PARN in complex with oligo(A)_3_ RNA (20) and superimposed them onto the human CCR4-CAF1 complex (Supplementary Figure S3E, F). Furthermore, to model an oligo(A)_2_ RNA substrate in the active site of CCR4, we additionally placed and fitted the penultimate nucleotide, based on the orientation of the corresponding desoxyribose in the complex of CCR4b with oligo(A)_3_ DNA (29). Finally, the orientation of the terminal two nucleotides in the active site of CAF1 is also supported by a more recent crystal structure of the PAN2 homolog from *S. cerevisiae* in complex with oligo(A)_5_ RNA (83), but the active site of human PARN is structurally more similar to CAF1 than the one of PAN2.

The models not only show the orientation of the terminal RNA nucleotides in the context of the residues that are generally conserved in EEP proteins (N195, E240, H361, D412, N414, T480, D491, S529, D530 and H531, Supplementary Figure S1A) and DEDDh nucleases (D40, E42, D161, D230 and H225, Supplementary Figure S1B), but also in the context of possibly more specialized structural elements. For CCR4, these are the apparently malleable ‘HWDP’-loop, which is critically positioned between the terminal two nucleotides, and the β-hairpin loop L(β18–β19) including N481, which may play a role in recognizing the RNA 3′-terminus (Figure 2A, C and Supplementary Figure S1A). For CAF1, these are S112, H157, H225 and the C-terminal tail (Figure 2B, D and Supplementary Figure S1B). Residues S112 and H225 are in a position to recognize the terminal base, but H225, assisted by H157, could also act in specialized catalysis. Apparently flexible, H157 and H225 flank the phosphoribose backbone from opposing sides and could act in multiple geometrically distinct combinations, dependent on their protonation state. Finally, the C-terminal tail of CAF1a can take a position to interfere with RNA substrate binding, as it was previously shown to occupy the active site cleft in a structurally defined manner (Figure 2B) (48).

### The CCR4 and CAF1 3′-exonucleases contribute distinctively to deadenylation by the CCR4-CAF1 complex

To investigate the enzymatic properties and substrate specificity of the crystallized human CCR4-CAF1 complex, as for crystallization, we purified the complex or its respective variants to homogeneity (Supplementary Figure S4, Table S2). To preserve nuclease activity, we strictly followed a highly standardized protocol, avoiding metal-chelating resins and the respective affinity tags. Consequently, separate preparations of the complex generated highly reproducible results in deadenylation time-course experiments. We started out from standard reaction conditions (50 mM HEPES at pH = 7.5, 150 mM NaCl, 2 mM MgCl_2_ and 1 mM DTT at 37°C), very similar to those originally established for the *S. cerevisiae* and human CCR4 proteins (29,84,85). RNA substrates (27-mers) were chosen to contain a 5′-FAM-labelled RNA body of seven nucleotides followed by a 3′-oligo(A) tail of twenty adenines. The RNA body either exclusively consisted of pyrimidines (Y_7_: UCUUCCU) or also contained additional adenines (N_7_: UCUAAAU) (29,84–86), resulting in the oligos ^5F^Y_7_A_20_ or ^5F^N_7_A_20_, respectively (Table S3). Deadenylation time-course experiments typically ran for 60 min and contained equimolar concentrations of 600 nM RNA substrate and 600 nM CCR4-CAF1 complex in order to simulate a situation where the two enzymes have been recruited to an mRNA via the CCR4-NOT complex and possibly compete for the single RNA 3′-end. These concentrations are considerably above cellular concentrations, which are estimated at approximately 100 nM for bulk mRNA (87) and less than 10 nM for the components of the CCR4-NOT complex (88).

**Figure 3.**
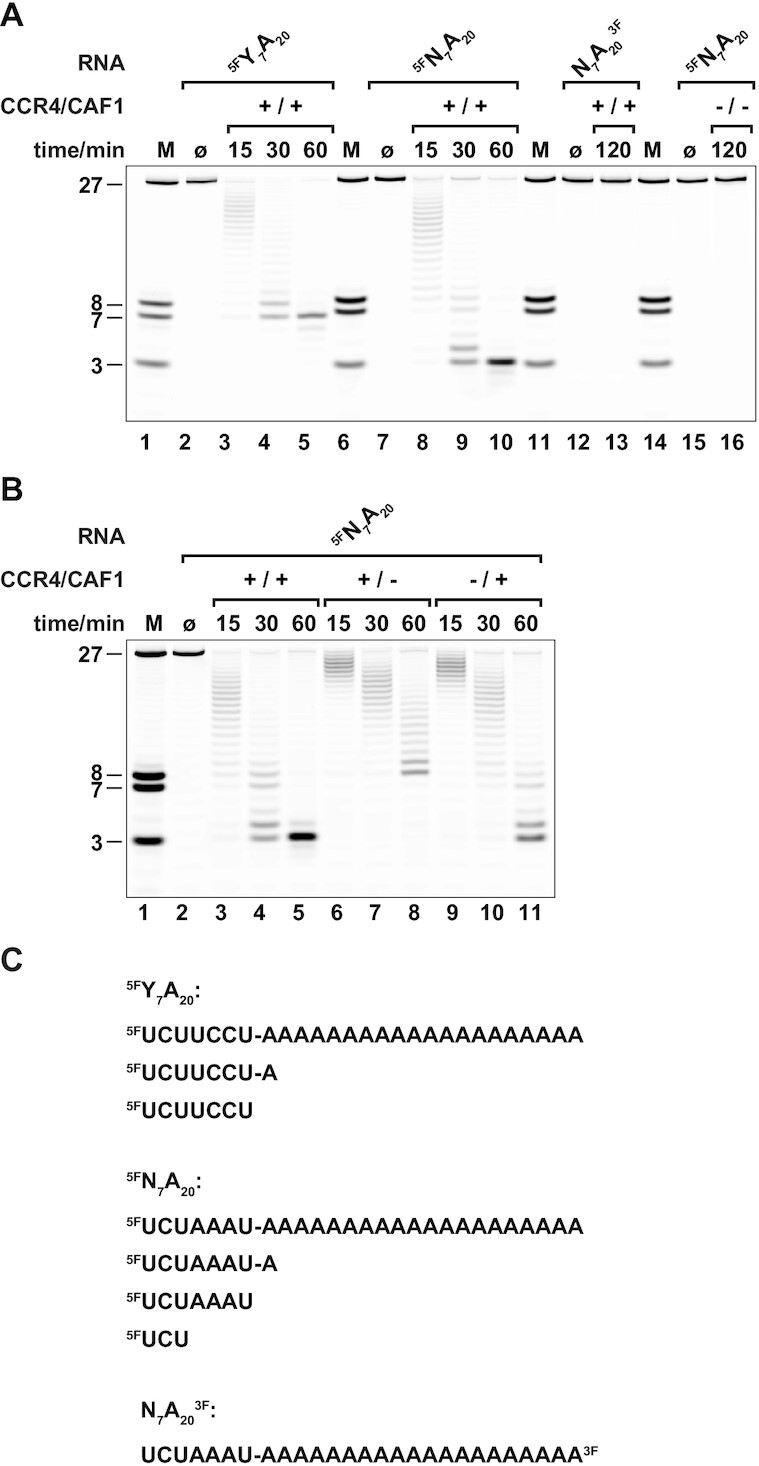
Activity and specificity of the CCR4 and CAF1 nucleases in the CCR4-CAF1 complex. (**A**) RNA 3′-deadenylation time course profiles using distinct RNA substrates. CCR4-CAF1 (+/+) deadenylates 5′-FAM-labeled 27-mer RNA molecules (^5F^Y_7_A_20_ and ^5F^N_7_A_20_). The 5′-RNA body does either lack (Y_7_: UCUUCCU) or contain additional adenine residues (N_7_: UCUAAAU). CCR4-CAF1 does not degrade a 3′-FAM-labeled RNA substrate (N_7_A_20_^3F^) and also lacks endonuclease activity. Active site point mutations of CCR4 (D491A) and CAF1 (D40A and E42A) completely inactivate RNA hydrolysis by CCR4-CAF1 (−/−). (**B**) Nuclease-specific deadenylation profiles. Active site point mutation of either CAF1 (+/−) or CCR4 (−/+) in the CCR4-CAF1 complex leads to distinguishable reaction products. CCR4 (+/−) stops one adenine short of the RNA body at ^5F^N_7_A, whereas CAF1 (−/+) continues into the RNA body of the ^5F^N_7_A_20_ substrate to stop only at ^5F^UCU. Initial deadenylation rates by CCR4 (+/−) and CAF1 (−/+) are comparable under standard reaction conditions. (**C**) RNA substrates and major products. Deadenylation assays were done under standard reaction conditions (0.6 μM CCR4-CAF1 complex and RNA substrate at 37°C in a buffer containing 50 mM HEPES at pH = 7.5, 150 mM NaCl, 2 mM MgCl_2_ and 1 mM DTT). Reaction products are resolved on denaturing (urea) gels with marker lanes (M) containing RNA oligonucleotides corresponding to the 27 nt substrates or to the most prominent products of RNA hydrolysis with sizes of 8 nt, 7 nt and 3 nt.

Taking standard conditions, the wildtype CCR4-CAF1 (+/+) complex progressively degraded ^5F^Y_7_A_20_ and ^5F^N_7_A_20_ RNA (Figure 3A, lanes 1–10, C), whereas a double mutant CCR4-CAF1 (−/−) complex (Table S2) with active site mutations in CCR4 (D491A, Figure 2A and Supplementary Figure S1A) (29,85) and CAF1 (D40A and E42A, Figure 2B and Supplementary Figure S1B) (79,86) was inactive and served as a negative control (Figure 3A, lanes 14–16, C). When the fluorescent label was placed at the 3′-end of the RNA substrate (N_7_A_20_^3F^, Table S3), the oligomer remained intact in the presence of the wildtype CCR4-CAF1 (+/+) complex (Figure 3A, lanes 11–13, C). Apparently, the 3′-label blocks 3′-exonucleolytic decay of the RNA substrate (Figure 3A, lane 13). Furthermore, we can conclude that our complex preparations lack endonuclease activity and also are free of other, contaminating nucleases (Figure 3A, lanes 13 and 16).

Interestingly, in the case of the ^5F^N_7_A_20_ RNA substrate, the 3′-exonucleolytic decay continued into the RNA body, leaving a final ^5F^UCU trinucleotide product (Figure 3A, lanes 9–10, C), whereas in the case of the ^5F^Y_7_A_20_ RNA substrate, RNA decay ended with a ^5F^UCUUCCU heptanucleotide after all of the adenines had been removed (Figure 3A, lanes 4–5, C). Consequently, at least one of the two nucleases must be able to remove 3′-terminal non-A nucleotides quite efficiently, such as the U7 nucleotide in the case of the ^5F^N_7_A_20_ RNA substrate.

We therefore prepared CCR4-CAF1 complexes where only one of the two nucleases was mutated at a time and tested them on ^5F^N_7_A_20_ RNA (Figure 3B, C). With the CCR4-CAF1 (+/−) complex, the reaction ended with a ^5F^UCUAAAUA octanucleotide product, one nucleotide short of the RNA body (Figure 3B, lanes 6–8, C), whereas with the CCR4-CAF1 (−/+) complex, the reaction continued up to the ^5F^UCU trinucleotide (Figure 3B, lanes 9–11, C). The progression of RNA decay into the RNA body of the ^5F^N_7_A_20_ RNA substrate is hence predominantly due to CAF1 and can also be taken as an indicator for the presence of CAF1 activity in the wildtype CCR4-CAF1 (+/+) complex. Because it reveals the distinctive contributions of the two enzymes, we hereafter chose ^5F^N_7_A_20_ RNA as our standard substrate in deadenylation experiments.

### The turnover of deadenylation by the CCR4-CAF1 complex remains concentration-dependent up into the micromolar range

The enzymatic turnover of CCR4 and CAF1 is approximately equal when acting on the oligo(A) tail under standard assay conditions. Judging from the midpoint of the ladder (Figure 3B, lanes 7 and 10), the rate of hydrolysis can be estimated at around 0.3 adenines per minute for either the CCR4-CAF1 (+/−) or the CCR4-CAF1 (−/+) complex and approximately twice as high for the wildtype CCR4-CAF1 (+/+) complex (Figure 3B, lane 3). These numbers are several orders of magnitude below the turnover of commercially available exonucleases such as of the non-processive EEP enzyme ExoIII (∼150 nt/ min, MCLab) or of the processive DEDD enzyme ExoI (∼3000 nt/ min, MCLab) (89), prompting us to further explore the dependence of deadenylation on the concentration of the CCR4-CAF1 complex and on complex formation between CCR4 and CAF1.

The increased turnover of the CCR4-CAF1 (+/+) complex as compared to the partially inactivated CCR4-CAF1 (+/−) and CCR4-CAF1 (−/+) complexes (Figure 3B) could be either a consequence of mass action (i.e. due to doubling the number of active sites at a concentration well below the saturation limit, resulting in a larger fraction of bound RNA) or a consequence of synergy (i.e. due to an interdependence or cooperation of active CCR4 and CAF1 in a common complex resulting in accelerated turnover), or a consequence of both mass action and synergy combined.

To distinguish between these possibilities (Supplementary Figure S5), we first tested for mass action by doubling the concentration of the ^5F^N_7_A_20_ RNA substrate or/and the CCR4-CAF1 (+/+) complex in deadenylation time course experiments (Supplementary Figure S5A). Doubling the RNA concentration did not shift the deadenylation profile (Supplementary Figure S5A, lanes 6–8 versus 3–5 and lanes 12–14 vs 9–11). This means that turnover doubled for each of the active sites, resulting in twice the rate of RNA hydrolysis in the reaction volume. Conversely, doubling the concentration of the CCR4-CAF1 complex also doubled the rate of RNA hydrolysis in the reaction volume (Supplementary Figure S5A, lane 9 versus 4 and lane 12 versus 7). Apparently therefore, we are operating at sub-saturating concentrations, and mass action can largely explain why deadenylation by the CCR4-CAF1 (+/+) complex is faster than deadenylation by the CCR4-CAF1 (+/−) or CCR4-CAF1 (−/+) complexes. The intrinsic affinity of the CCR4-CAF1 complex for RNA 3′-ends is sufficiently low for the deadenylation rate to respond proportionally to changes in the concentration of the complex, even when the latter reaches values up to and beyond one micromolar.

To test for synergy between CCR4 and CAF1 upon complex formation, we performed a mixing experiment, where the concentration of active sites and RNA substrate remained at a constant value (Supplementary Figure S5B). Mixing wildtype CCR4-CAF1 (+/+) complex with an equal concentration of inactivated CCR4-CAF1 (−/−) complex did not shift the deadenylation profile (Supplementary Figure S5B, lanes 9–11 versus 3–5). With a mixture of CCR4-CAF1 (+/−) and CCR4-CAF1 (−/+) complexes, however, deadenylation was detectably slower than with a mixture of CCR4-CAF1 (+/+) and CCR4-CAF1 (−/−) complexes (Supplementary Figure S5B, lanes 6–8 versus 9–11), suggesting some synergy when the active enzymes interact within a complex. Although the observed effect is clearly less than twofold and probably can already be explained by the avidity arising from the proximity of the two active sites in the CCR4-CAF1 complex, there likely are also additional benefits of complex formation, such as mutual effects on the binding geometry of the RNA substrate (see below and Figure 8A).

In summary, these data are consistent with a model for CCR4-CAF1, where both nucleases contribute about equally to deadenylation and operate as strict 3′-exonucleases that remove nucleotides one-by-one and with turnover limited by substrate binding for probably each reaction cycle. Considering cellular concentraions of less than 10 nM for CCR4 and CAF1, their recruitment to mRNA targets by additional factors is essentially obligatory for an efficient mRNA deadenylation and decay. This results in numerous possibilities for a specific, quantitative and combinatorial regulation, as the overall rate of deadenylation will depend on the number, the nature, and the quality of individual recruitment sites within a given mRNA molecule.

### The relative contributions of CCR4 and CAF1 strongly vary with pH

**Figure 4.**
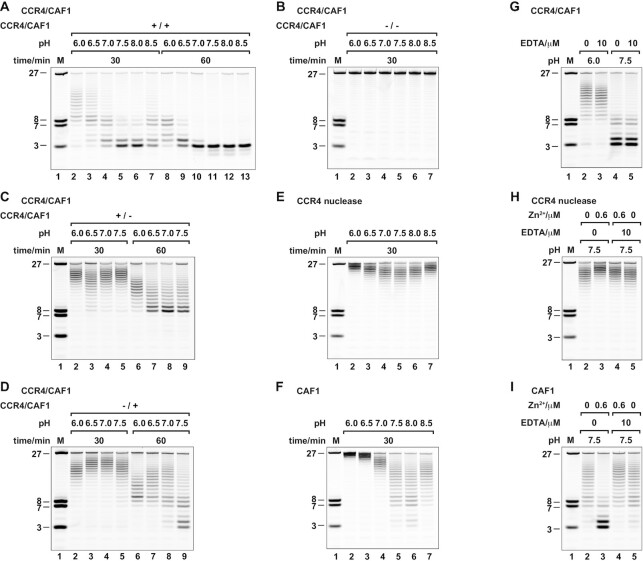
Differential modulation of CCR4 and CAF1 nuclease activity by pH and zinc ions in stoichiometric amounts. (**A, B**) pH-dependence of the CCR4-CAF1 complex. Deadenylation occurs over a wide range of pH (A) and critically depends on the integrity of the active sites (B). (**C, D**) pH-dependence of the individual nucleases within the CCR4-CAF1 complex. Hydrolysis by CCR4 (+/−) occurs over a much narrower pH range than observed in panel (A) with an optimum at neutral to slightly acidic pH (C). Hydrolysis by CAF1 (−/+) shows a complex pH-dependence with apparently two pH optima at moderately acidic and moderately basic pH (D). (**E, F**) pH-dependence of the isolated individual CCR4 and CAF1 nucleases. Hydrolysis by the isolated CCR4 nuclease domain shows a single pH optimum at around neutral pH (E). Hydrolysis by the isolated CAF1 shows a single pH optimum at moderately basic pH (F), lacking the activity at moderately acidic pH observed in the context of inactivated CCR4 (D). Compare also Wang *et al.* (29) and Bianchin *et al.* (86) for initial characterizations of isolated human CCR4 nuclease (pH = 7.4) and isolated CAF1 (pH = 8.0), respectively. (**G–I**) Specific stimulation of CAF1 activity by stoichiometric quantities of zinc ion. Standard reaction conditions (containing 2 mM of magnesium ion) are not affected in the presence of 10 μM EDTA, used here as a strong zinc ion chelator (G, control). The isolated CCR4 nuclease domain (0.6 μM) is slightly inhibited by the addition of stoichiometric amounts of zinc ion (0.6 μM) to standard reaction conditions (H). The isolated CAF1 nuclease is clearly stimulated under the same conditions (I). DTT was omitted from (H) and (I) to avoid interference from its capacity to also act as a zinc ion chelator (see also Supplementary Figure S6).

Under standard reaction conditions, CCR4 and CAF1 contribute about equally to deadenylation by the CCR4-CAF1 complex (Figure 3B). Because both enzymes are metal-dependent and have titratable histidines in their active centers (Figure 2A, B and Supplementary Figure S2C, D), we tested their response to changing pH and divalent metal ion concentrations (Figure 4 and Supplementary Figure S6).

We found deadenylation by the wildtype CCR4-CAF1 (+/+) complex to work efficiently over a very wide range of pH, with an optimum at a moderately basic pH of around 7.5–8.0 (Figure 4A), whereas the inactivated CCR4-CAF1 (−/−) complex did not cause any RNA hydrolysis over the whole range of pH conditions (Figure 4B). The pH profiles of the partially inactivated CCR4-CAF1 (+/−) and CCR4-CAF1 (−/+) complexes however differed to a great extent (Figure 4C, D), resulting in variable pH-dependent contributions to deadenylation.

Whereas the complexed CCR4 shows a rather ‘normal’ pH profile with a single optimum at a neutral pH of around 6.5–7.0 (Figure 4C), the complexed CAF1 shows a highly unusual, ‘inverted’ pH profile (Figure 4D). Complexed CAF1 performs worst at a neutral pH of 6.5–7.0, with two distinct optima at or above a pH of 7.5 and at or below a pH of 6.0.

Moreover, at the lower pH optimum, CAF1 acts more slowly on shorter substrates than at the higher pH optimum and/or discriminates better against non-A nucleotides (Figure 4D, lanes 2 and 6 vs 5 and 9). This is evident as well with the wildtype CCR4-CAF1 (+/+) complex (Figure 4A–D, lanes 2 vs 5). Consequently, the activities and contributions of CCR4 and CAF1 are differentially affected by pH, not only in quantitative but also in qualitative terms.

### Deadenylation by CAF1 is modulated by the interaction with CCR4

A dual pH optimum with distinct substrate specificity is highly unusual for an enzyme with a single active site. To investigate whether the complicated pH-dependence of CAF1 is an inherent property of its active site or whether it arises from CCR4-CAF1 complex formation, we determined the pH-dependence of the isolated CCR4 and CAF1 nucleases and compared it to the results obtained from the partially inactivated CCR4-CAF1 (+/−) and CCR4-CAF1 (−/+) complexes.

In the case of the isolated CCR4 nuclease domain, the pH profile is very similar to the one of the complexed CCR4 protein with only a modest downshift of the optimal pH within the neutral range upon inclusion in the complex (Figure 4E versus C). In contrast, the pH profile of the isolated CAF1 nuclease is clearly different from the one of the complexed CAF1 nuclease (Figure 4F vs D). Isolated CAF1 shows a single pH optimum at the moderately basic pH of 7.5–8.0 and only poor activity at the moderately acidic pH of 6.0. This suggests that CAF1 activity at the lower pH optimum is a consequence of complex formation.

We therefore conclude that complex formation between CCR4 and CAF1 not only provides a kinetic advantage, but also endows the complex with qualitatively distinct properties that are more than the sum of its parts (see also Supplementary Figure S5B). Apparently, complexed CAF1 can bind RNA substrates in multiple ways and/or has the ability, via its active site harboring two titratable histidines (H157 and H225, Figure 2B, D and Supplementary Figure S1B), to catalyze reactions that are geometrically or chemically distinct.

### Deadenylation by CAF1 is strongly responsive to the concentration of Mg^2+^ and trace amounts of Zn^2+^

Cellular concentrations of free Mg^2+^ are usually estimated at less than 2 mM (90), and metal-dependent enzymes are usually optimized for best performance with Mg^2+^ concentrations in the low millimolar range, showing little sensitivity to small changes in ion concentration. The CCR4-CAF1 (+/+) complex, as expected, was completely inactive in the absence of Mg^2+^, which was assured by the presence of 10 mM EDTA as an effective chelator of divalent metal ions (Supplementary Figure S6A, lane 2) (91). Surprisingly however, deadenylation was strongly responsive and stimulated by Mg^2+^ concentrations above 2 mM, with an optimum concentration of apparently more than 10 mM under standard conditions (Supplementary Figure S6A, lanes 3–5).

The steep response to the change in Mg^2+^ concentration can be assigned to CAF1, as tested in the context of the CCR4-CAF1 (−/+) complex (Supplementary Figure S6A, lanes 10–13), whereas CCR4, as tested in the context of the CCR4-CAF1 (+/−) complex (Supplementary Figure S6A, lanes 6–9), already gets inhibited at Mg^2+^ concentrations of more than 2 mM. This unusual Mg^2+^-dependent stimulation of CAF1 also occurs with the isolated nuclease, and both at the moderately acidic pH of 6.0 and at the standard pH of 7.5, although the enzyme activity remains poor at the lower pH (Supplementary Figure S6B).

Considering the sensitivity of deadenylation by CAF1 to the concentration of Mg^2+^, and considering that the activity of the CAF1 homolog from *Schizosaccharomyces pombe* (*S. pombe*) was previously found modulated by trace metal ions (75 μM Mn^2+^, 220 μM Zn^2+^) (92), we also examined CAF1 for this possibility. Indeed, and in contrast to the CCR4 nuclease, we found CAF1 to be stimulated already by sub-micromolar concentrations of Zn^2+^, equal to and below the concentration of CAF1 (600 nM) (Figure 4G–I and Supplementary Figure S6C, D).

The Zn^2+^-dependent stimulation potently occurs both under moderately acidic and under standard reaction conditions (Supplementary Figure S6C) and is further enhanced in the absence of DTT (Supplementary Figure S6D), which is otherwise routinely present in our reactions as a protective reducing agent and at a concentration of 1 mM. At this concentration, however, DTT also acts as a potent chelator and ligand of Zn^2+^ (93,94) and, if omitted, unmasks the effect of Zn^2+^ on CAF1 activity (Figure 4H, I and Supplementary Figure S6D).

We therefore conclude that CAF1 is an ion-sensitive enzyme stimulated by stoichiometric amounts of Zn^2+^ (Figure 4I, lanes 2–3). This is different from CCR4 (Figure 4H, lanes 2–3) and suggests that CAF1 works at suboptimal speed in the cell and/ or that the contribution of CAF1 to deadenylation is tunable by pH and the availability of Mg^2+^ and Zn^2+^. For comparative conclusions, it is hence essential to precisely define and control experimental conditions. In the present case, we verified the absence of Zn^2+^ from standard assay conditions by repeating experiments in the presence of 10 μM EDTA (Figure 4G), which efficiently removes traces of Zn^2+^ in the presence of 2 mM Mg^2+^ (Figure 4H, I, lanes 4–5 versus 2–3) (93,94), and we strictly kept Mg^2+^ at a concentration of precisely 2.0 mM.

### Deadenylation is assisted by the CCR4 LRR domain and moderated by the CAF1 C-terminal tail

**Figure 5.**
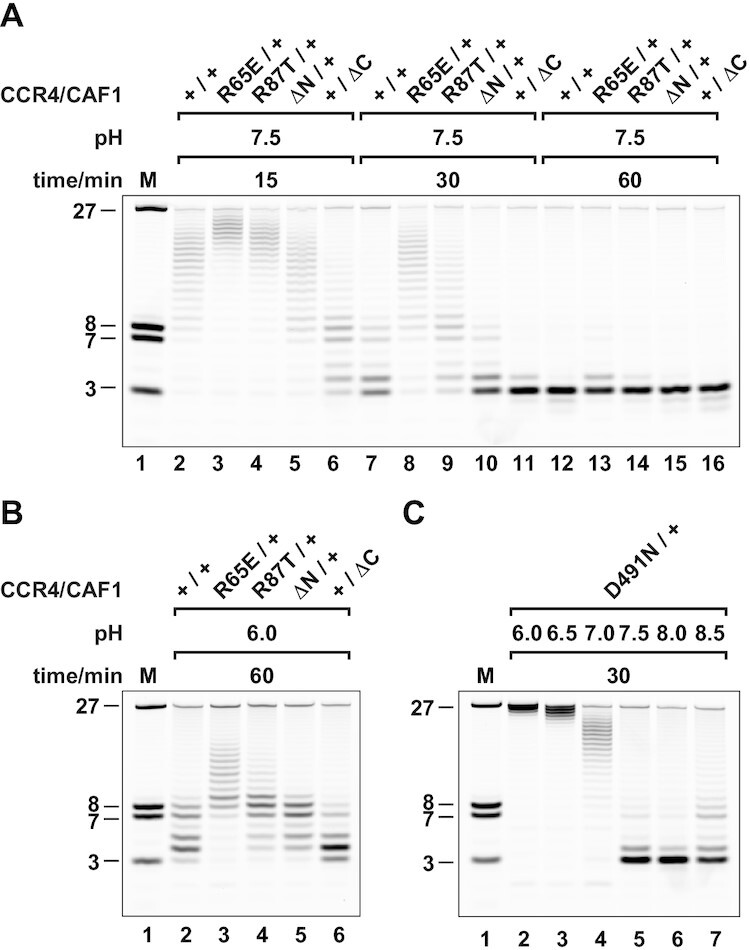
Structure-based mutational analysis of the CCR4-CAF1 complex. (**A**) Effects of peripheral (non-active site) mutations on deadenylation by CCR4-CAF1 under standard reaction conditions (pH = 7.5). Replacement of positively charged surface residues on the CCR4 LRR domain with residues corresponding to the *Sc* CCR4 LRR domain delays deadenylation (R65E/+ or R87T/+). Deletion of the CCR4 N-terminal extension does not have a detectable effect (ΔN/+), whereas deletion of the CAF1 C-terminal tail facilitates deadenylation (+/ΔC). (**B**) Effects of peripheral (non-active site) mutations on deadenylation by CCR4-CAF1 under moderately acidic reaction conditions (pH = 6.0). Results are comparable to (A) without differential effects on either one of the nucleases. (**C**) Differential effect of a CCR4 active site mutation on deadenylation by CAF1. In contrast to a D491A mutation of the CCR4 active site (Figure 4D, –/+), a D491N mutation causes the CCR4-CAF1 complex to lose activity at moderately acidic pH and to display a pH dependence comparable to the isolated CAF1 nuclease (Figure 4F).

To investigate whether and how CCR4-CAF1 complex formation modulates access of the RNA substrate to the individual active sites, we generated a series of structure-based CCR4-CAF1 complex variants and compared their deadenylation profiles (Figure 5).

In a first step, we mutated single residues on the positively charged surface of the human CCR4 LRR domain. We tested an R65E and an R87T mutation (R65E/+ and R87T/+, Table S2), converting poorly defined arginine side chains to the residues found in *S. cerevisiae*, where the CCR4 LRR domain is rather neutrally charged (Figure 1G, H and Supplementary Figures S1A and S2E–H). We found deadenylation activity of the CCR4-CAF complex to be reduced with each of the two variants, and down to roughly half the rate in the case of the R65E mutation (Figure 5A, B). This suggests that the CCR4 LRR domain of the human CCR4-CAF1 complex indeed assists and guides mRNA substrate binding.

In a second step, we also tested whether the very N-terminal region of CCR4 (M1–K28) and the very C-terminal tail of CAF1 (G263–S285) matter for the deadenylation activity of the CCR4-CAF1 complex (Figure 1A and Supplementary Figure S1), although the amino acid sequences of these regions are not conserved in non-vertebrates. The deletion of residues M1–K28 from the N-terminus of CCR4 (ΔN/+, Table S2) did not affect deadenylation detectably (Figure 5A, B), although the crystal structure suggests residues Y6–N25 to participate in the fixation of loop L(β2–α2) of CAF1 (Figure 1C and Supplementary Figure S2A, C). The deletion of residues S264–S285 from the C-terminal tail of CAF1 (+/ΔC, Table S2), however, almost doubled the deadenylation rate (Figure 5A, B). Although they are disordered in the present crystal structure, residues G274–E280 were previously found to occlude the active site of CAF1 (Figure 2B, D) (48). Upon complex formation with CCR4, they might consequently constrain the space for the RNA to access the active site cleft of CAF1 (Supplementary Figures S2A, C and S3A, B).

### The CCR4 nuclease domain modulates deadenylation by CAF1

The deadenylation profiles obtained with the CCR4 LRR point mutations or with the terminal deletion variants continued to show contributions of both nucleases (Figure 5A, B). In particular, also the complex-dependent deadenylation activity of CAF1 persisted, which is most evident at a pH of 6.0 and reflected by an eventual shortening of the ^5F^N_7_A_20_ RNA substrate beyond nucleotide U7 (Figure 5B).

Surprisingly however, in combination with a D491N active site mutation of CCR4 (D491N/+, Figure 2A and Supplementary Figure S1A, Table S2), CAF1 essentially lost all of its activity at the lower pH (Figure 5C) and behaved just like the isolated CAF1 nuclease alone (Figure 4F). The D491N variant (D491N/+) was designed to alter the hydrogen bonding network in the active site of the CCR4 nuclease domain and unlikely affects complex formation between the CCR4 LRR domain and CAF1. Nevertheless, the status of the flexibly linked CCR4 nuclease domain apparently affects the activity at the active site of CAF1. The CCR4 nuclease domain may hence participate in the control of RNA substrate binding to the CCR4-CAF1 complex in a complex-dependent manner, but how this is precisely achieved currently remains unclear.

### Deadenylation by CCR4-CAF1 requires an unobstructed 3′-hydroxyl group on the terminal nucleotide

**Figure 6.**
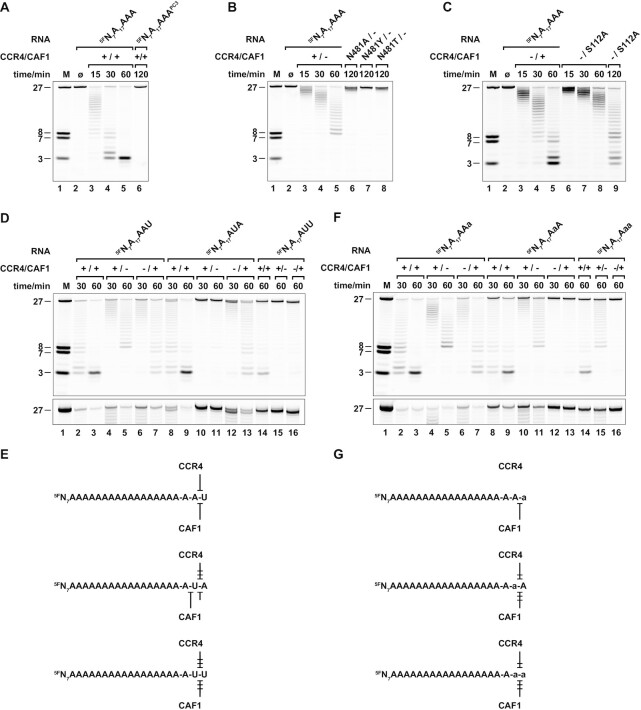
Specificity of RNA 3′-end recognition by the CCR4-CAF1 complex. (**A–C**) Importance and recognition of an unobstructed 3′-hydroxyl group. Already the presence of a 3′-terminal phosphate (included as a propionyl phosphate and denoted PC3) completely prevents hydrolysis of the ^5F^N_7_A_20_ RNA substrate, denoted here as ^5F^N_7_A_17_AAA (A). Structure-based point mutations support a direct role of CCR4 N481 (B) and CAF1 S112 (C) in the recognition of the 3′-hydroxyl group. The CCR4-CAF1 complex (+/+) or the complex with individually inactivated CAF1 (+/−) or CCR4 (−/+) nucleases is indicated as before. (**D, E**) Importance of nucleobase identity on both sides of the scissile phosphate. Uridines were introduced in the terminal (^5F^N_7_A_17_AAU) or penultimate (^5F^N_7_A_17_AUA) positions of the oligonucleotide substrate or in both of these positions (^5F^N_7_A_17_AUU). The panel (D) shows a comparative gel with samples selected and reorganized from Supplementary Figure S7. See panels (A-C) for the unmodified RNA substrate ^5F^N_7_A_17_AAA. In case of the ^5F^N_7_A_17_AUA substrate, note the presence of double bands in the 2-fold vertical zoom onto the 27 nt marker region at the bottom of the panel. The scheme (E) highlights phosphodiester linkages where the rate of hydrolysis by CCR4 or CAF1 is reduced, and where the number of blunt arrowheads indicates the extent of the reduction. (**F, G**) Importance of the 2′-hydroxyl group on both sides of the scissile phosphate. Deoxyadenines are indicated by lowercase letters and were introduced in the terminal (^5F^N_7_A_17_AAa) or penultimate (^5F^N_7_A_17_AaA) positions of the oligonucleotide substrate or in both of these positions (^5F^N_7_A_17_Aaa). The panel (F) shows a comparative gel with samples selected and reorganized from Supplementary Figure S7. See panels (A–C) for the unmodified RNA substrate ^5F^N_7_A_17_AAA. The scheme (G) highlights phosphodiester linkages where the rate of hydrolysis by CCR4 or CAF1 is reduced, and where the number of blunt arrowheads indicates the extent of the reduction.

Because the two enzymes apparently work in a distributive and randomly alternating fashion, they must perform equally well in recognizing an RNA 3′-end and in avoiding endonucleolytic degradation of the RNA body. We hence attached a small propyl-phosphate (a monophosphate-monopropylester) to the RNA 3′-end in order to simulate the continuation of the phosphodiester backbone (^5F^N_7_A_17_AAA^PC3^, Table S3). The presence of the propyl-phosphate essentially blocked deadenylation by CCR4-CAF1 (Figure 6A). Both enzyme active sites consequently require an unobstructed 3′-hydroxyl group at the terminal ribose for hydrolysis to proceed, and probably a precise positioning of the 3′-terminal nucleotide via hydrogen bonds, as suggested by previous crystal structures (20,29,48).

For CCR4 as an EEP enzyme, the terminal adenine would be unstacked from the preceding nucleotide and accommodated in a specific pocket, as observed for an AMP molecule in complex with the CCR4b nuclease (29). In this pocket, the Hoogsteen edge (95) of the adenine is contacted by the backbone carbonyl group of N412, with the side chain of N481 on the β-hairpin loop L(β18–β19) in hydrogen bonding distance to the ribose 3′-hydroxyl group and blocking the space for a continued phosphoribose backbone (Figure 2A, C and Supplementary Figure S3E). N481 is conserved among CCR4 homologs (Supplementary Figure S1A), but not among other EEP enzymes (32,33). We therefore replaced N481 with a bulky tyrosine to block the space for the 3′-terminal ribose (N481Y/+, Table S2), or with an alanine to prevent hydrogen bond formation (N481A/+, Table S2). In both cases, deadenylation was blocked almost completely when tested in combination with the CAF1 active site mutant (Figure 6B, lanes 6, 7). Deadenylation was not even supported by a threonine (Figure 6B, lane 8), which is observed at the equivalent position of the closely related nocturnin proteins (N481T/+, Table S2) (28,96,97). We consequently propose N481 to play a specialized role in the context of CCR4 proteins, in recognizing the RNA 3′-end and in positioning the terminal nucleotide for catalysis.

For CAF1 as a DEDDh enzyme, the terminal adenine is likely orientated as observed in the related PARN nuclease, where both the 2′-hydroxyl group and the 3′-hydroxyl group of the terminal ribose are recognized and fixed via hydrogen bonds to the protein backbone of β-strand β2, and without leaving space for a continuation of the RNA chain. Furthermore, the side-chain of S112, which is conserved both in CAF1 and in PARN proteins (Supplementary Figure S1B) (20), is in a position for a hydrogen bond with either the 2′- hydroxyl group or the N3 atom on the sugar edge (95) of the terminal adenine (Figure 2B, D and Supplementary Figure S3F). We consequently generated an S112A mutant in complex with the CCR4 active site mutant (−/S112A, Table S2) and found that deadenylation was considerably slower (Figure 6C), as previously observed in the context of the CAF1 homolog from *S. pombe* (92). This observation indicates that CAF1 indeed recognizes a 3′-terminal adenine in a fashion that is highly similar to PARN.

### Nucleobase identity affects hydrolysis by CCR4 or CAF1 from both sides of the scissile phosphate

Concluding from the previous experiments, the strict 3′-exonuclease activity of both CCR4 and CAF1 relies on the precise positioning of the 3′-terminal ribose moiety. Furthermore, the respective models for terminal nucleotide recognition suggest a selectivity for adenine via specific hydrogen bonds to the Hoogsteen edge of the nucleobase in the case of CCR4 and to the sugar edge in the case of CAF1, but not to the Watson–Crick edge (95) of the adenine, which would provide the highest degree of ‘positive’ selectivity.

To explain adenine specificity, one therefore needs to consider also ‘negative’ selectivity, i.e. a discrimination against non-A nucleotides. Negative selectivity could arise from steric clashes of functional groups, resulting in a catalytically unfavorable geometry of the phosphoribose backbone upon binding. Moreover, due to differences in nucleotide stacking geometry, the backbone between non-A nucleotides may be less well predisposed for a kinetically efficient interaction. Extending to both sides of the scissile phosphate, these effects can reduce the need for a specific recognition of nucleobases via hydrogen bonds and especially for the penultimate nucleotide position in DEDDh deadenylases, for which such positive selection is apparently lacking (20,83).

We therefore modified the terminal and the preceeding residues in a series of oligonucleotide substrates and tested them with the wildtype CCR4-CAF1 (+/+) complex as well as with the individually inactivated CCR4-CAF1 (+/−) and CCR4-CAF1 (−/+) complexes (Figure 6D–G and Supplementary Figure S7). Substituting the terminal three adenines by uridines (^5F^N_7_A_17_UUU, Table S3) simulates an mRNA 3′-uridinylation (61–63) and essentially prevented 3′-nucleotide hydrolysis by the CCR4-CAF1 complex (Supplementary Figure S7B vs S7A). This result is consistent with the stop of nucleotide hydrolysis at the transition to the RNA body of the ^5F^Y_7_A_20_ substrate (Figure 3A, lanes 1–5, C). With the ^5F^N_7_A_20_ substrate, however, nucleotide hydrolysis continued into the RNA body (Figure 3A, lanes 6–10, C), because the single uridine at position 7 was insufficient to stop the CAF1-dependent reaction (Figure 3B, lanes 9–11, C). Similarly, a substitution of only the terminal adenine (^5F^N_7_A_17_AAU, Table S3) delayed deadenylation by the CCR4-CAF1 complex, but also did not stop the reaction (Figure 6D, lanes 2–7, 6E and Supplementary Figure S7C vs S7A). Remarkably however, not only the CCR4-CAF1 (−/+) complex (Figure 6D, lanes 6–7, E), but also the CCR4-CAF1 (+/−) complex hydrolyzed the ^5F^N_7_A_17_AAU substrate quite efficiently (Figure 6D, lanes 4–5, 6E), whereas in the ^5F^N_7_A_20_ substrate, the single uridine was sufficient to stop the CCR4-dependent reaction (Figure 3B, lanes 6–8, C).

The puzzle was resolved with the ^5F^N_7_A_17_AUA substrate (Table S3), with the uridine in the penultimate position (Figure 6D, lanes 8–13, 6E and Supplementary Figure S7D vs S7A). In this case, the removal of the terminal adenine by the CCR4-CAF1 (+/−) complex was strongly impaired (Figure 6D, lanes 10–11, E). This observation also explains the stop of the CCR4-CAF1 (+/−) complex one adenine short of the RNA body of the ^5F^N_7_A_20_ substrate (Figure 3B, lane 8, C). The penultimate uridine also reduces terminal adenine hydrolysis by the CCR4-CAF1 (−/+) complex, but less strongly than in the case of the CCR4-CAF1 (+/−) complex (Figure 6D, lanes 12–13 versus 10–11, E). Furthermore, in case of the CCR4-CAF1 (−/+) complex, we observe a characteristic double band caused by the additional delay of deadenylation by the uridine in the terminal position of the ^5F^N_7_A_17_AU decay intermediate (Figure 6D, lanes 12–13, E). Finally, with two consecutive uridines at the 3′-end (^5F^N_7_A_17_AUU, Table S3), both of the effects combine, efficiently preventing even CAF1 from hydrolyzing a terminal uridine (Figure 6D, lanes 14–16, E and Supplementary Figure S7E vs S7A).

### Deadenylation by CCR4-CAF1 depends on the geometry and integrity of the RNA backbone

The terminal adenine substitution experiments clearly show the importance of nucleotide identity on both sides of the scissile bond, but from the modeled substrates alone (Figure 2C, D), the mechanism of how nucleotide identity is ‘sensed’ in the penultimate position remains unclear. In an effort to assess the importance of a defined backbone geometry around the scissile bond, we therefore tested additional substrates, where the 2′-hydroxyl group was removed from the terminal ribose, from the penultimate ribose, or from both the terminal and penultimate ribose (Figure 6F, G and Supplementary Figure S7F–H).

Consistent with the proposed mode of terminal nucleotide recognition, the removal of the 2′-hydroxyl group (^5F^N_7_A_17_AAa, Table S3) did not slow down the CCR4-dependent reaction of the CCR4-CAF1 (+/−) complex, but it modestly delayed deadenylation by the CCR4-CAF1 (−/+) complex (Figure 6F, lanes 2–7, G and Supplementary Figure S7F vs S7A). The removal of the 2′-hydroxyl group from the penultimate adenine (^5F^N_7_A_17_AaA, Table S3), however, strongly impaired the CAF1-dependent deadenylation, and also the CCR4-dependent reaction was clearly affected (Figure 6F, lanes 8–13, G and Supplementary Figure S7G versus S7A). Finally, with the removal of the 2′-hydroxyl groups from both the terminal and penultimate nucleotide (^5F^N_7_A_17_Aaa, Table S3), the negative effects on deadenylation again combined (Figure 6F, lanes 14–16, G and Supplementary Figure S7H versus S7A).

We conclude that even minor changes to mRNA substrate binding kinetics or to the precise phosphodiester binding geometry have considerable impact on the efficiency of terminal nucleotide hydrolysis. Furthermore it is conceivable that, in the case of CAF1, the 2′-hydroxyl group of the penultimate nucleotide even participates directly in the chemical step of the reaction, judging from the crystal structures of PARN (20) and the PAN2 homolog (83) in the presence of RNA and from our derived model (Figure 2D and Supplementary Figure S3F).

### Non-A residues delay deadenylation by CCR4-CAF1 in a nucleotide-specific manner

**Figure 7.**
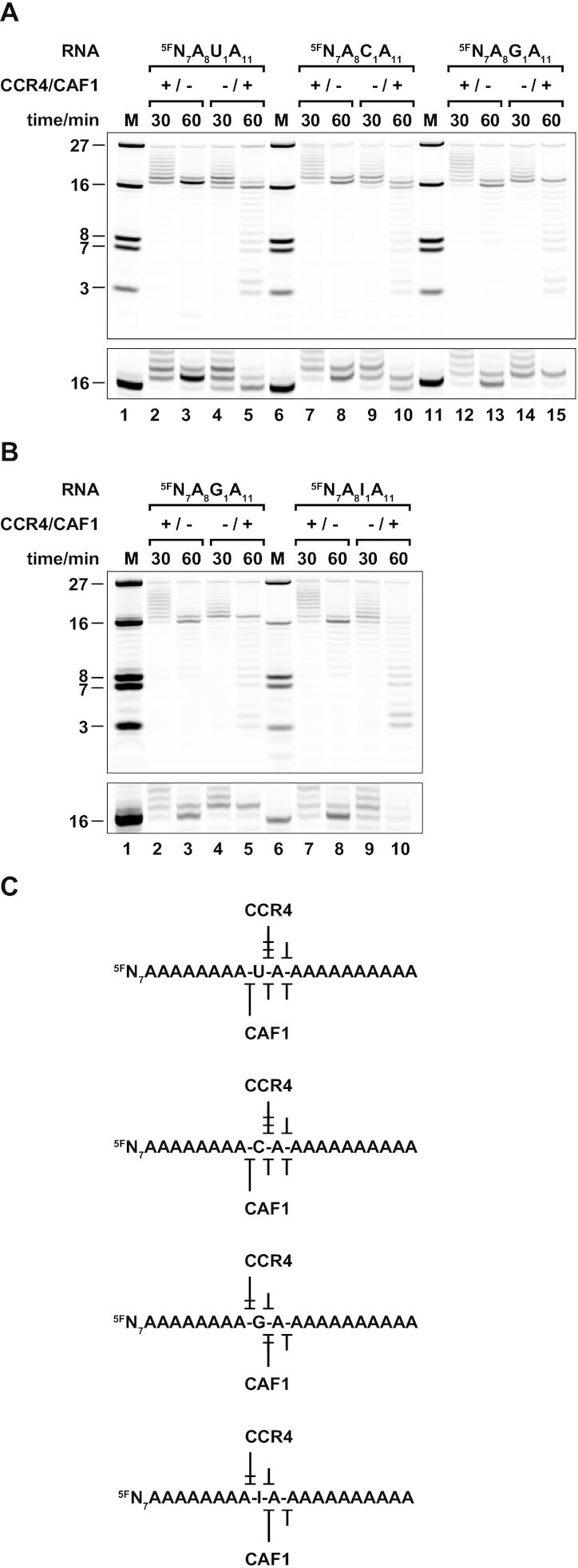
Differential effect of interspersed non-A nucleotides on deadenylation by the CCR4-CAF1 complex. (**A**) Discrimination against non-A nucleotides in the active sites of CCR4 (+/−) and CAF1 (−/+). Position A16 of the ^5F^N_7_A_20_ RNA substrate was substituted with a pyrimidine (uridine or cytidine; substrate denoted as ^5F^N_7_A_8_UA_11_ or ^5F^N_7_A_8_CA_11_) or another purine (guanine; substrate denoted as ^5F^N_7_A_8_GA_11_). (**B**) Discrimination against inosine. Position A16 of the ^5F^N_7_A_20_ RNA substrate was substituted with an inosine, the direct deamination product of adenine (substrate denoted as ^5F^N_7_A_8_IA_11_). Panels (A) and (B) show comparative gels with samples selected and reorganized from Supplementary Figure S8. Note the diversity and the precise position of each of the primary product bands in the 2-fold vertical zoom onto the 16 nt marker region at the bottom of each panel. Importantly, and depending on the individual structural context, the non-A nucleotides are frequently rate-limiting already before they reach the 3′-terminal position in the RNA substrate, occasionally even from the pre-penultimate position. (**C**) Effects of non-A nucleotides on deadenylation. The scheme highlights phosphodiester linkages where the rate of hydrolysis by CCR4 or CAF1 is reduced, and where the number of blunt arrowheads indicates the extent of the reduction.

Finally, we also investigated the effect of other possible nucleotides and how they affect deadenylation. To this aim and for better nucleotide resolution, we systematically substituted the adenine at the internal position 16 of the ^5F^N_7_A_20_ substrate (Figure 7 and Supplementary Figure S8).

In agreement with the results from the 3′-terminal nucleotide substitutions, an A16U substitution (^5F^N_7_A_8_U_1_A_11_, Table S3) blocked deadenylation by the CCR4-CAF1 (+/−) complex already when the uridine reached the penultimate position, whereas deadenylation by the CCR4-CAF1 (−/+) complex was affected much less and predominantly after the uridine had reached the terminal position (Figure 7A, lanes 2–5, C and Supplementary Figure S8A). A careful analysis of the deadenylation profiles reveals an effect of the uridine even already from the pre-penultimate position, suggesting that both enzyme active sites are able to ‘sense’ the identity of up to three consecutive nucleotides at the 3′-end of an mRNA. A very similar result was obtained with an A16C substitution (^5F^N_7_A_8_C_1_A_11_, Table S3), only that the cytidine caused minimally stronger effects than the uridine (Figure 7A, lanes 7–10, C and Supplementary Figure S8B). This result suggests that each of the two enzymes handles pyrimidines in a similar way and does not distinguish between the functional groups at the C4 position of the pyrimidine base. The A16G substitution (^5F^N_7_A_8_G_1_A_11_, Table S3), however, had an entirely different outcome. In this case, the CCR4-dependent reaction was affected only after the guanine had reached the terminal position, and it was not blocked as strongly as with the substrates containing a pyrimidine. In contrast, now the CAF1-dependent reaction was affected more strongly than with the substrates containing a pyrimidine, and already when the guanine was in the penultimate position (Figure 7A, lanes 12–15, C and Supplementary Figure S8C). Apparently therefore, pyrimidine and purine nucleotides are inversely recognized by each of the two enzymes, and in a position-dependent manner.

This interpretation is supported by a last experiment where A16 was substituted by an inosine (^5F^N_7_A_8_I_1_A_11_, Table S3), which naturally results from the spontaneous or enzymatic deamination of an adenine (98). The A16I substitution showed deadenylation profiles very similar to the ones obtained from the A16G substitution, and the CCR4-dependent reaction was similarly affected when the inosine reached the 3′-terminal position on the RNA substrate. This observation suggests a read-out via the Hoogsteen edge (95), such as illustrated by the crystal structure of CCR4b with AMP (29). The CAF1-dependent reaction was however less strongly affected when the guanine was replaced by the inosine, possibly because the inosine lacks the exocyclic amino group at position C2 on the purine base and allows a more favorable backbone geometry (Figure 7B, lanes 7–10 versus 2–5, C and Supplementary Figure S8D).

We conclude that both CCR4 and CAF1 clearly discriminate against non-A residues at the RNA 3′-end, sensing up to three consecutive nucleotides. In combination with a distributive mode of action, this allows the CCR4-CAF1 complex to rapidly remove the 3′-poly(A) tail from an RNA while overcoming single non-A nucleotides, and nevertheless to stop when the RNA body is reached.

## DISCUSSION

### Significance of the human CCR4-CAF1 crystal structure

The crystal structure of the human CCR4-CAF1 complex is an important advance towards understanding the molecular framework and specificity of mRNA deadenylation in metazoan species. In comparison to the previously determined but only partially complete structure of the complex from *S. cerevisiae* (36), the present structure helps to identify and localize common and adaptive features. This comparison is particularly useful, because the two deadenylases from *S. cerevisiae* are rather only distantly related to their metazoan homologs (Supplementary Figure S1), and even fungal sequences from *S. pombe* have higher sequence identity to the human homologs (CCR4, 43.1% and CAF1, 57.8%) than they have to the sequences from *S. cerevisiae* (CCR4, 39.9% and CAF1, 40.2%).

Despite the low sequence identity, it is therefore reassuring that the general position of the individual protein domains in the structures of the human and *S. cerevisiae* complexes is very similar, and that the mode of CCR4 attachment to CAF1 via the CCR4 LRR domain is maintained. Furthermore, in both structures, the CCR4 nuclease domain is suspended over the helical side of the CCR4 LRR domain, but the orientation differs by approximately 80 degrees. This strongly suggests a generally flexible linkage with the LRR domain. Whether and how the relative position of the two nucleases adapts in the context of the CCR4-NOT complex therefore remains to be seen, but neither in the orientation of the *Hs* CCR4 nuclease domain, nor in the orientation of the *Sc* CCR4 nuclease domain is it possible for the two active sites of CCR4 and CAF1 to sufficiently approach each other for working jointly together on the 3′-end of a bound mRNA. Finally, the *Sc* CCR4-CAF1 complex clearly differs from its human homolog by the presence of a long and partially structured N-terminal extension on *Sc* CCR4, by the absence of the positively charged patch found on the surface of the human CCR4 LRR domain, by the molecular details of the interface between the CCR4 LRR domain and CAF1, and by substitutions of catalytic residues in the active site of CAF1 (Figure 1 and Supplementary Figures S1 and S2).

The crystal structure of the human CCR4-CAF1 complex (CCR4a-CAF1a) also improves our current molecular and mechanistic understanding of the CCR4 and CAF1 paralogs that exist in vertebrate species, and in particular of CCR4a, which had not been crystallized before. Paralog-specific differences rather map to peripheral regions of the proteins, and comparative structure analysis of paralogs and crystal forms identifies malleable elements of likely functional importance (Supplementary Figure S1 and S3).

In a larger perspective, the present structure of the human CCR4-CAF1 complex will also help to model the catalytic core and its conformation(s) in the context of the CCR4-NOT complex, and it will serve to stimulate and interpret future experiments also in this extended context.

### Distinct recognition of RNA 3′-ends by CCR4 and CAF1

The presence of two distinct deadenylases in the CCR4-NOT complex with entirely different protein architecture suggests CCR4 and CAF1 to serve non-redundant functions in mRNA deadenylation. Nevertheless, for a reliable and efficient deadenylation, each nuclease must meet a number of partially conflicting requirements, which equally apply to both enzymes.

First of all, both nucleases specifically need to recognize 3′-terminal adenines and efficiently remove them, but strictly without causing endonucleolytic cleavage at internal oligo(A) sequences, as this would jeopardize cellular mRNA integrity. Second, both enzymes need the ability to remove occasional non-A nucleotides from the poly(A)-tail that result from spurious or mRNA regulatory processes in the cell (64–67), but they also need to stop 3′-exonucleolytic mRNA decay when they reach the mRNA body and after the poly(A)-tail is removed. Consequently, and third, both CCR4 and CAF1 need to recognize 3′-terminal adenines as a part of an extended array of adenines, but without delaying product release or compromising on the speed of deadenylation turnover.

In the case of CAF1, the substrate nucleotides are thought to be recognized in a stacked conformation, such as observed in PARN and PAN2 (20,83) and similar to the presumed conformation of oligo(A) RNA in solution, which is thought to resemble the A-form geometry known from double-stranded RNA (99,100). As a DEDDh enzyme, CAF1 uses its DEDD residues to coordinate two metal ions that flank two sides of the scissile phosphate (82,101), whereas the conserved but flexible histidine H225 (Supplementary Figure S1) (35,48,102) is thought to help generate the nucleophile and/or participates in fixing the terminal nucleotide (20,103–105). Moreover, histidine H157, which is exclusively conserved in CAF1 enzymes (Supplementary Figure S1) (35), is also thought to participate in substrate binding (105) and may flexibly contact the backbone of the penultimate residue for a precise fixation, possibly in a pH-dependent manner (Figure 2 and Supplementary Figure S3).

In the case of CCR4, the terminal nucleotide is thought to be bound in an unstacked conformation, flipped away from the preceding nucleotides and recognized as observed in the structure of the human CCR4b nuclease (29). As an EEP enzyme, CCR4 may operate with only a single metal ion, but also fix the RNA backbone in a precise orientation (33,80,81). We identified asparagine N481 as a crucial residue for an efficient catalysis, which is exclusively conserved in CCR4 enzymes (Supplementary Figure S1) (32,33) and poised to recognize the RNA 3′-hydroxyl group on the terminal nucleotide. Furthermore, the HWDP-loop emerges as a highly conserved but apparently malleable loop that may participate in the fixation of the penultimate nucleotide (Figure 2 and Supplementary Figure S3).

In both enzyme active sites, the modeled substrates leave no space to continue the RNA chain beyond the 3′-terminal nucleotide, which is confirmed by our results where already the presence of a 3′-terminal phosphate completely blocked hydrolysis (Figures 2 and 6). This binding mode assures that both enzymes exclusively work in an exonucleolytic mode.

Our results also show that single non-A nucleotides within or at the 3′-end of the poly(A) tail all cause a local delay of nucleotide hydrolysis, but that they are eventually removed for deadenylation to proceed (Figures 6 and 7 and Supplementary Figures S7 and S8). This removal is primarily mediated by CAF1, as it acts more rapidly on the respective substrates. Importantly however, the non-A residues are ‘detected’ not only in the terminal position of the oligonucleotide substrates. Dependent on enzyme type and nucleotide identity, the non-A residues can delay deadenylation considerably already from the penultimate position of the substrate, or even from the pre-penultimate position, leaving one or two 3′-terminal adenines on the respective deadenylation product. Consequently, with the occurrence of several consecutive non-A nucleotides the effects combine factorially, essentially stopping nucleotide removal at the transition from the mRNA poly(A)-tail to the mRNA body, whereas single non-A nucleotides can be used by the cell to diversify the deadenylation and decay of individual mRNA molecules (65,67,106). Oligo(A)-substrates with several consecutive non-A nucleotides at their 3′-end eventually can also be degraded by CCR4-CAF1. This was reported for a *Drosophila melanogaster* (*D. melanogaster*) CCR4-CAF1 complex, when used in an approximately 1000-fold excess over the RNA substrate (107).

Finally, it remains a perplexing question how CAF1 and CCR4 manage to ‘scan’ consecutive nucleotides at an RNA 3′-end without generating numerous adenine-specific hydrogen bonds that would considerably delay product release and general turnover times. We therefore favor a model, where hydrolysis is affected also by indirect effects of non-A nucleotides on the precise geometry of the RNA phosphodiester backbone. Indeed, it was recently reported that nucleotide-specific base-stacking interactions predispose the backbone geometry of an oligo(A)-substrate for an efficient hydrolysis by DEDDh nucleases such as PAN2 and CAF1 (83). Non-A nucleotides would consequently disturb base-stacking interactions and RNA backbone geometry over a distance of up to several nucleotides and especially if their functional groups clash with portions of the enzyme scaffold. Unperturbed oligo(A)-substrates, however, would efficiently pass this filter, and without incurring a kinetic penalty that would result from the positive recognition of multiple adenines by numerous and specific hydrogen bonds.

### The versatility of CAF1 in mRNA 3′-deadenylation

**Figure 8.**
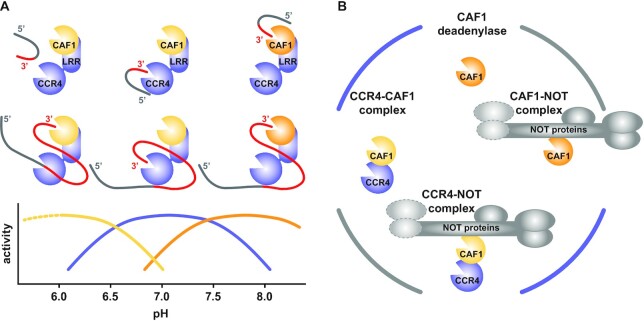
Versatility of CAF1 in mRNA 3′-deadenylation. (**A**) Possible modes of substrate binding and enzyme activity. At moderately basic pH, CAF1 (orange color) can work independently of CCR4, but the positively charged patch on the CCR4 LRR domain may facilitate binding of the RNA substrate (grey with poly(A) tail in red). At moderately acidic pH, activity of CAF1 (yellow color) depends on the presence of CCR4, and the active site of the CCR4 nuclease domain likely participates in RNA substrate binding. CCR4 (blue) works best at neutral pH, complementing the activity of CAF1. Substrate binding modes vary with RNA length and pH. (**B**) Evolutionary aspects and modes of operation. CAF1 deadenylase: CAF1 (orange) might have served as a primordial deadenylase. It is tunable by the ionic environment and apparently active individually in certain cells and circumstances. CAF1 complex with CCR4: Flexibly tethered CCR4 (blue) extends the catalytic range of associated CAF1 (now yellow). Furthermore, the catalytic activity of CCR4 complements CAF1 in the complex, increasing the efficiency of nucleotide hydrolysis with certain substrates and facilitating deadenylation in the presence of PABPC1 (9,10). CAF1 complex with NOT proteins: The NOT proteins (grey) serve as an adaptor, assuring the efficient recruitment of CAF1 (yellow) to selected mRNAs. Fully assembled CCR4-NOT complex: the fully assembled CCR4-NOT complex controls deadenylation by CAF1 and CCR4 in the context of selective mRNA regulation and decay.

One of the most curious aspects regarding the human CCR4-NOT complex is its evolutionary history and the question of why, in addition to its centrally embedded CAF1 nuclease, it contains a second and more peripherally attached CCR4 nuclease. We therefore investigated reaction parameters and substrate recognition for the individual nucleases and how they change upon CCR4-CAF1 complex formation (Figures 3–5 and Supplementary Figures S5 and S6). This analysis supports a picture of CAF1 as a highly versatile and tunable deadenylase, dependent on environmental conditions and interaction partners. CAF1 gets assisted by CCR4 to improve deadenylation efficiency with a subset of substrates and over a wider range of environmental conditions, and associates with the NOT proteins and/or auxiliary factors for the purpose of selective mRNA targeting and regulation (Figure 8).

We observe that human CAF1 can act independently of associated CCR4 at moderately basic pH, whereas at moderately acidic pH and for sufficiently long RNA substrates, CAF1 activity depends on cooperation with CCR4 (Figures 4, 5 and 8A). Although the details of RNA substrate binding and CAF1 catalysis at each of the two pH optima currently remain unclear, our observations may serve to partially reconcile apparently conflicting reports in the literature. In particular, Maryati *et al.* reported a strict interdependence of the two enzyme activities for the human complex (108), whereas others did not report any interdependence for the human (109), *D. melanogaster* (107) and *S. pombe* (110) complexes. Besides species-specific adaptations, variable ratios and excess of enzyme over substrate, or the presence of auxiliary factors such as BTG2 (108), clearly also the pH conditions can affect experimental results and conclusions here. In particular, Maryati *et al.* conducted their experiments at a pH of 7.9 (108), where we find deadenylation to be dominated by the CAF1-dependent reaction (Figure 8A), whereas Raisch *et al.* used a pH of 6.8 that was chosen for the comparison with the CCR4-NOT complex of *S. pombe* (109,110), and where we find the contribution of human CAF1 to be near a minimum (Figure 8A).

From an evolutionary perspective (Figure 8B), CAF1 as an independent and tunable single-domain enzyme, could well have served as a primordial deadenylase. In contrast to human CCR4, we find human CAF1 highly responsive to the ion concentration of Mg^2+^ and Zn^2+^ and possibly using its C-terminal tail to moderate activity (Figures 4, 5 and Supplementary Figure S6). The CCR4 nuclease domain could have originated from an ancient endonuclease that has acquired the LRR domain in order to associate with CAF1 (28). CCR4 is beneficial, because it complements the pH profile of CAF1 (Figure 8A) and prefers guanosines among non-A nucleotides, complementing the ability of CAF1 with pyrimidines to rescue deadenylation in the case of spurious or regulatory non-A residues within the mRNA poly(A) tail (Figure 7 and Supplementary Figure S8). Furthermore, and different from CAF1, CCR4 is described to hydrolyze poly(A) RNA that is covered by PABPC1 (9,10), possibly because CCR4 distorts the RNA backbone upon binding, whereas CAF1 prefers a regular RNA backbone geometry (Figure 2 and Supplementary Figure S3). CAF1 does however not depend on CCR4 to act on 3′-poly(A) tails in the presence of PABPC1, because deadenylation by CAF1 in the presence of PABPC1 is efficient if CAF1 associates with auxiliary factors from the BTG/TOB family (111,112). Finally, we found CCR4 to assist deadenylation via the positively charged surface of its LRR domain (Figures 5 and 8A). Very likely, this surface improves RNA substrate binding and possibly guides the RNA 3′-end for hydrolysis in either one of the active sites. In the context of the larger CCR4-NOT complex, additional modulations of enzyme activity are likely to occur, including a generally enhanced binding of mRNAs via additional RNA-binding surfaces (109,110,113,114), which also could alter the pH-dependence of RNA binding.

The association of CAF1 with the NOT proteins has likely evolved however not for improving general RNA binding, but for the specific recruitment of the deadenylases to selected mRNA targets (Figure 8B). Together with previous work (36,48,105,115,116), our present structure confirms that docking of the NOT proteins is possible in the presence of CCR4 and/or the APRO domain of BTG/TOB proteins, emphasizing the central structural role of CAF1 in the assembly (Supplementary Figure S9). As demonstrated also by structural analysis, mRNA selection can occur via the miRNA-induced silencing complex (miRISC) (45,46) or via specialized RNA-binding proteins that directly recruit the CCR4-NOT complex (117–120). Importantly here, the recruitment itself can already explain the accelerated deadenylation and decay of the targeted mRNAs, because even at enzyme and substrate concentrations of 600 nM, the turnover rate still is highly concentration-dependent (Supplementary Figure S5). As these concentations are at least one order of magnitude above physiological levels, regulating CCR4-NOT complex recruitment, and hence regulating the local deadenylase concentration, can directly regulate mRNA decay.

Interestingly however, CAF1 can also act independently of the CCR4-NOT complex. Most prominently, this is known from murine spermatogenesis, where CAF1a is essential (56,57) and found in an apparent complex with the MIWI protein and pachytene piRNAs. This piRNA-induced silencing complex (piRISC) is thought to target and eliminate most mRNAs from elongating spermatids (58). Moreover, also Zn^2+^ appears to be important in spermatogenesis and sperm capacitation (121,122), as well as in oocyte fertilization (123), such that the presently identified sensitivity of CAF1 to nanomolar concentrations of Zn^2+^ could indeed be physiologically meaningful (124).

The functional diversification of nuclease complexes via specialization, repurposing and secondary acquisition of structurally unrelated nucleolytic enzymes is not without precedence if one considers the evolution of the bacterial, archaeal and eukaryotic exosome complexes (34). From this perspective, the multitude of CAF1 and CCR4 homologs and paralogs in eukaryotic species can be regarded as an example of molecular evolution caught in the act. Accordingly, in *S. cerevisiae*, the CCR4 homolog probably carries out most of the mRNA deadenylation in the presence of a catalytically impaired CAF1 homolog (79,125), whereas on the other side of the spectrum CCR4a+CCR4b double knockout mice are viable (49) and there are certain ‘CCR4-NOT’ complexes from protists or plants that entirely seem to lack a CCR4 component (52,54).

In conclusion, we therefore consider CAF1 as an ‘amazing wizard’ that may continue to surprise with novel functionality. Assisted by CCR4 as its ‘assiduous partner’, it acts in a variety of cellular contexts that, in order to be investigated, require a very careful monitoring and control of experimental conditions. Given the presently uncovered subtleties, it will clearly be a challenge for future research to experimentally untangle the respective activities in a physiological context. Such work will be of crucial importance, however, for a detailed and molecular understanding of cellular RNA metabolism and its consequences on gene expression.

## DATA AVAILABILITY

Atomic coordinates and structure factors for the reported crystal structure have been deposited with the Protein Data Bank (126) under accession number 7ax1.

## Supplementary Material

gkab414_Supplemental_File

## Notes

Present address: Ying Chen, Department of Molecular Biology, Max Planck Institute for Biophysical Chemistry, Am Faßberg 11, D-37077 Göttingen, Germany.

Present address: Elena Khazina, CureVac AG, Friedrich-Miescher-Str. 15, D-72076 Tübingen, Germany.
