# Supplementary material for: Crystal structure and functional properties of the human CCR4-CAF1 deadenylase complex
Source: Nucleic Acids Res. 2021 May 26;49(11):6489–510. doi: 10.1093/nar/gkab414 (PMC8216464; doi:10.1093/nar/gkab414)
Supplement: gkab414_Supplemental_File [file gkab414_supplemental_file.pdf]

Crystal structure and functional properties of the human CCR4-CAF1 deadenylase complex

Ying Chen[#], Elena Khazina[#], Elisa Izaurralde[†] and Oliver Weichenrieder[*]

Department of Biochemistry, Max Planck Institute for Developmental Biology, Max-Planck-Ring 5, D-72076 Tübingen, Germany

[#] The authors wish it to be known that, in their opinion, the first 2 authors should be regarded as joint First Authors.

* To whom correspondence should be addressed. Tel: +49-7071-601-1358;

Email: oliver.weichenrieder@tuebingen.mpg.de

† deceased 30 April 2018

Present Address:

Ying Chen, Department of Molecular Biology, Max Planck Institute for Biophysical Chemistry, Am Faßberg 11, D-37077 Göttingen, Germany.

Present Address:

Elena Khazina, CureVac AG, Friedrich-Miescher-Str. 15, D-72076 Tübingen, Germany.

- SUPPLEMENTARY INFORMATION -

Supplementary Figures S1–S9

Supplementary Tables S1–S3

Supplementary References

Figure S1. Structure-based sequence alignments of CCR4 and CAF1 proteins

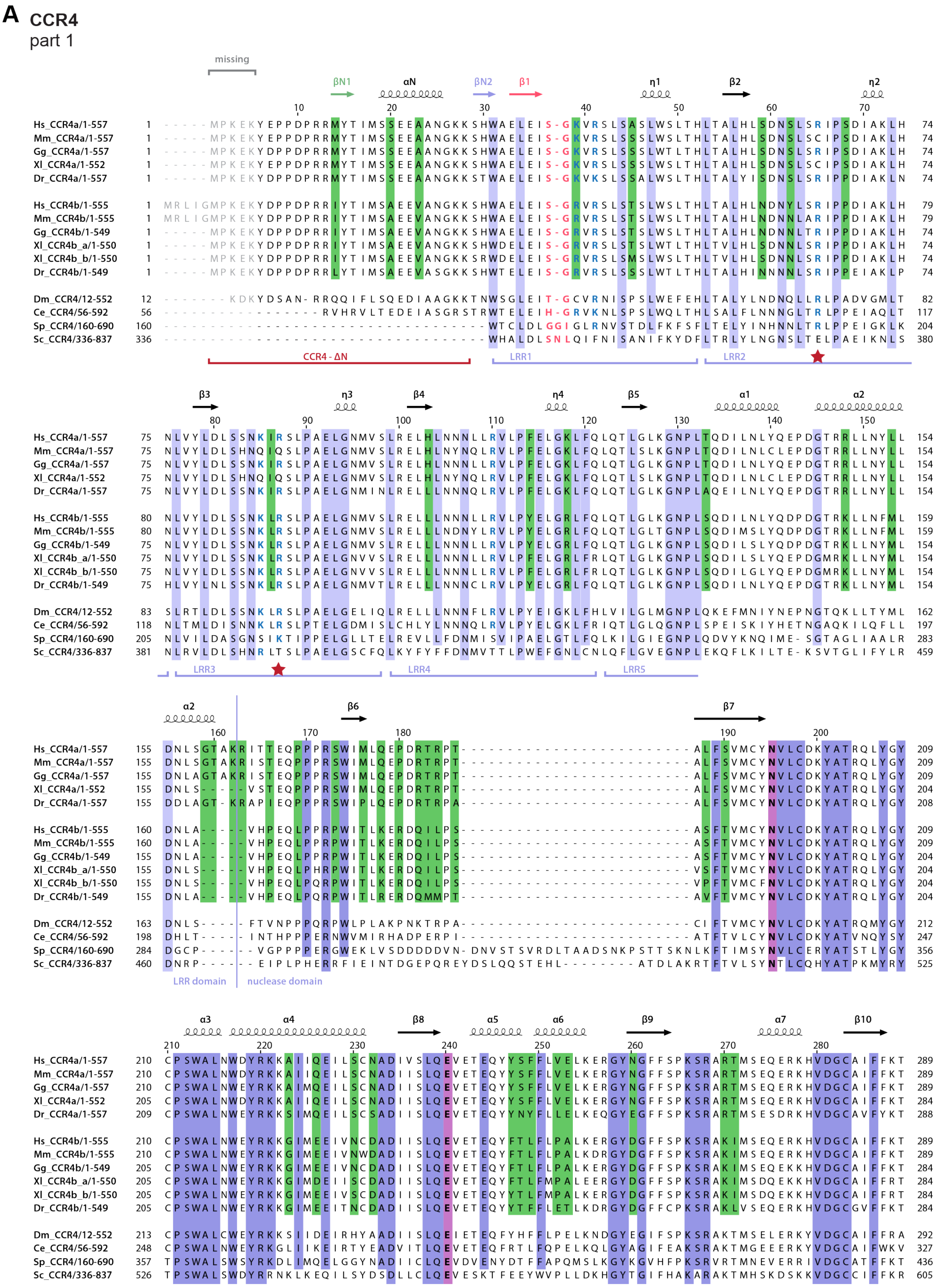

Figure S1. Structure-based sequence alignments of CCR4 and CAF1 proteins

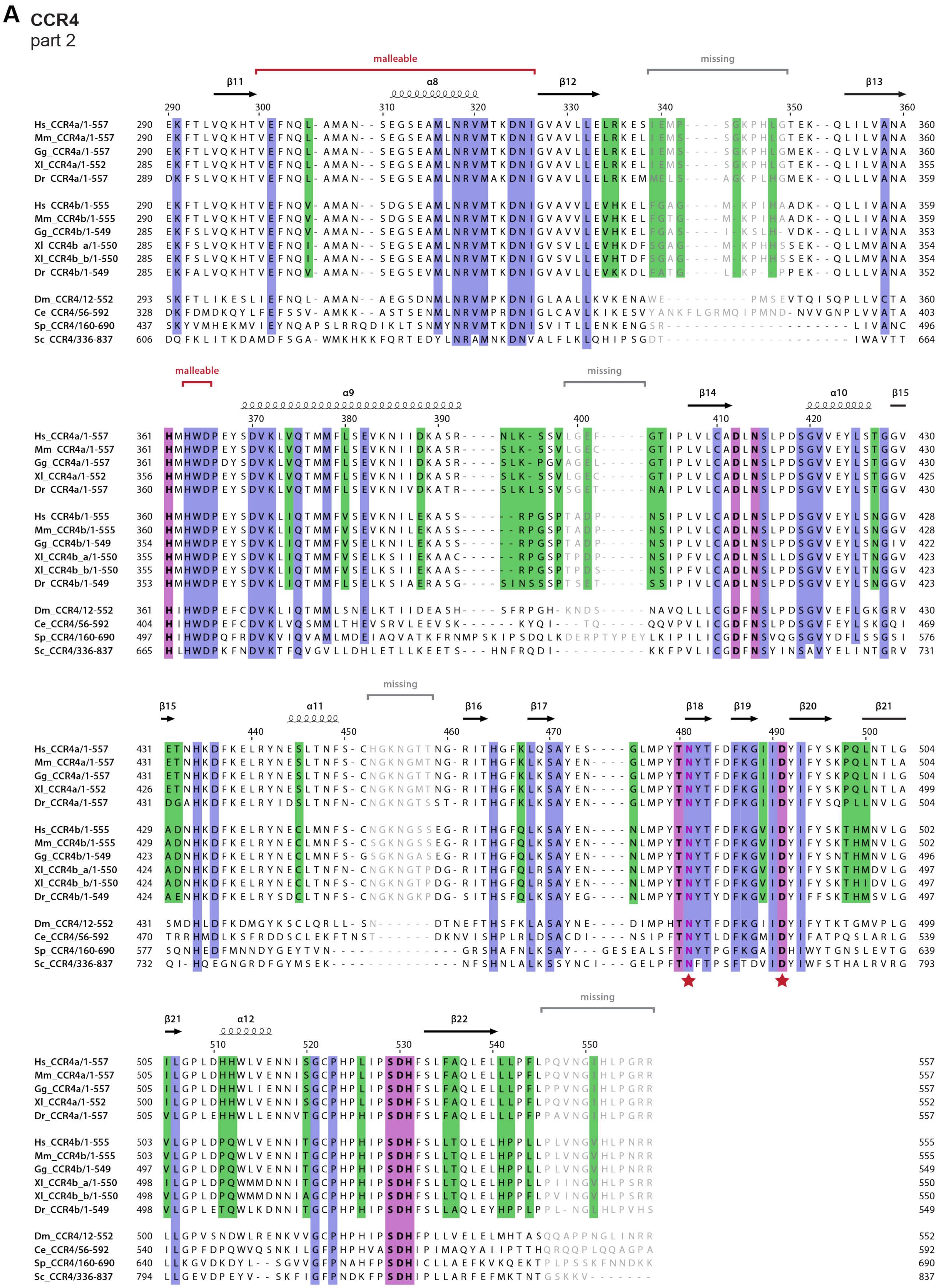

Figure S1. Structure-based sequence alignments of CCR4 and CAF1 proteins

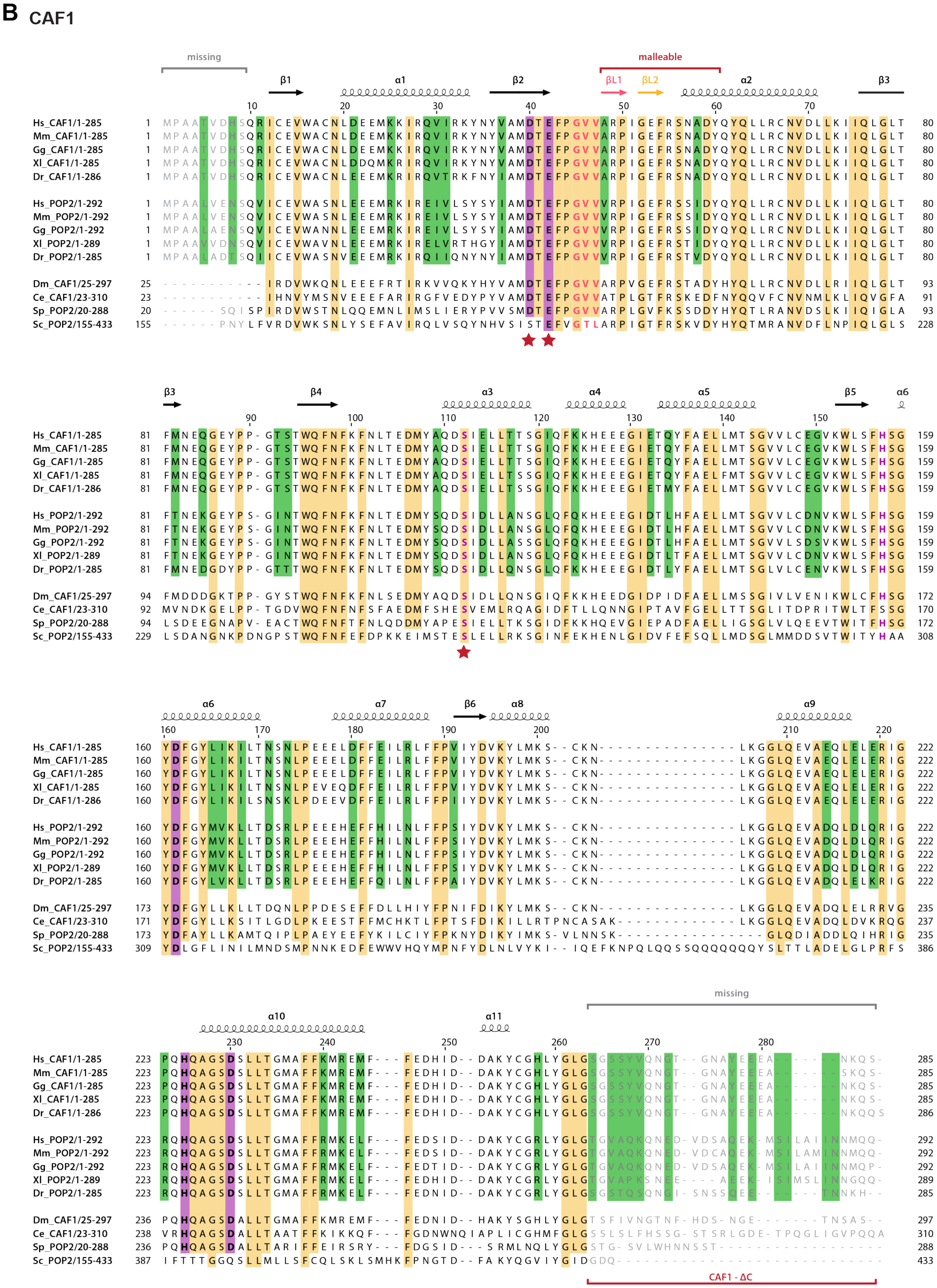

Figure S1. Structure-based sequence alignments of CCR4 and CAF1 proteins

(A) Alignment of CCR4 proteins. Vertebrate CCR4a and CCR4b paralogs are aligned with other metazoan and fungal CCR4 proteins. Secondary structure elements as found in the crystal structure of the *Hs* CCR4-CAF1 complex are indicated on top of the alignment, together with portions of the sequence that are missing from the structure or presumably malleable. Residues that are identical among all sequences with the exception of *Sc* CCR4 are shaded in blue (light blue for the LRR domain and slate blue for the nuclease domain) and residues defining the EEP nuclease family are shaded in purple. Positions that vary systematically between vertebrate paralogs are shaded in green. Residues that were deleted (Δ) or mutated (asterisk) in the course of this work are indicated below the alignment. Other important residues are highlighted in bold and colored purple for participating in RNA 3'-end recognition, blue for being part of the positively charged patch on the CCR4 LRR domain or salmon for relevance in the context of the interface between the CCR4 LRR domain and CAF1. Species abbreviations are as follows: *Hs*, *Homo sapiens*; *Mm*, *Mus musculus*; *Gg*, *Gallus gallus*; *Xl*, *Xenopus laevis*; *Dr*, *Danio rerio*; *Dm*, *Drosophila melanogaster*; *Ce*, *Caenorhabditis elegans*; *Sp*, *Schizosaccharomyces pombe*; *Sc*, *Saccharomyces cerevisiae*.

(B) Alignment of CAF1 proteins. Vertebrate CAF1a and CAF1b (POP2) paralogs are aligned with other metazoan and fungal CCR4 proteins. Secondary structure elements as found in the crystal structure of the *Hs* CCR4-CAF1 complex are indicated on top of the alignment, together with portions of the sequence that are missing from the structure or presumably malleable. Residues that are identical among all sequences with the exception of *Sc* CCR4 are shaded in yellow and residues defining the DEDDh nuclease family are shaded in purple. Positions that vary systematically between vertebrate paralogs are shaded in green. Residues that were deleted (Δ) or mutated (asterisk) in the course of this work are indicated below the alignment. Other important residues are highlighted in bold and colored purple for participating in RNA 3'-end recognition or salmon for relevance in the context of the interface between the CCR4 LRR domain and CAF1. Species abbreviations are as follows: *Hs*, *Homo sapiens*; *Mm*, *Mus musculus*; *Gg*, *Gallus gallus*; *Xl*, *Xenopus laevis*; *Dr*, *Danio rerio*; *Dm*, *Drosophila melanogaster*; *Ce*, *Caenorhabditis elegans*; *Sp*, *Schizosaccharomyces pombe*; *Sc*, *Saccharomyces cerevisiae*.

Figure S2. Structural details of CCR4-CAF1 complex formation and of the human CCR4 LRR domain

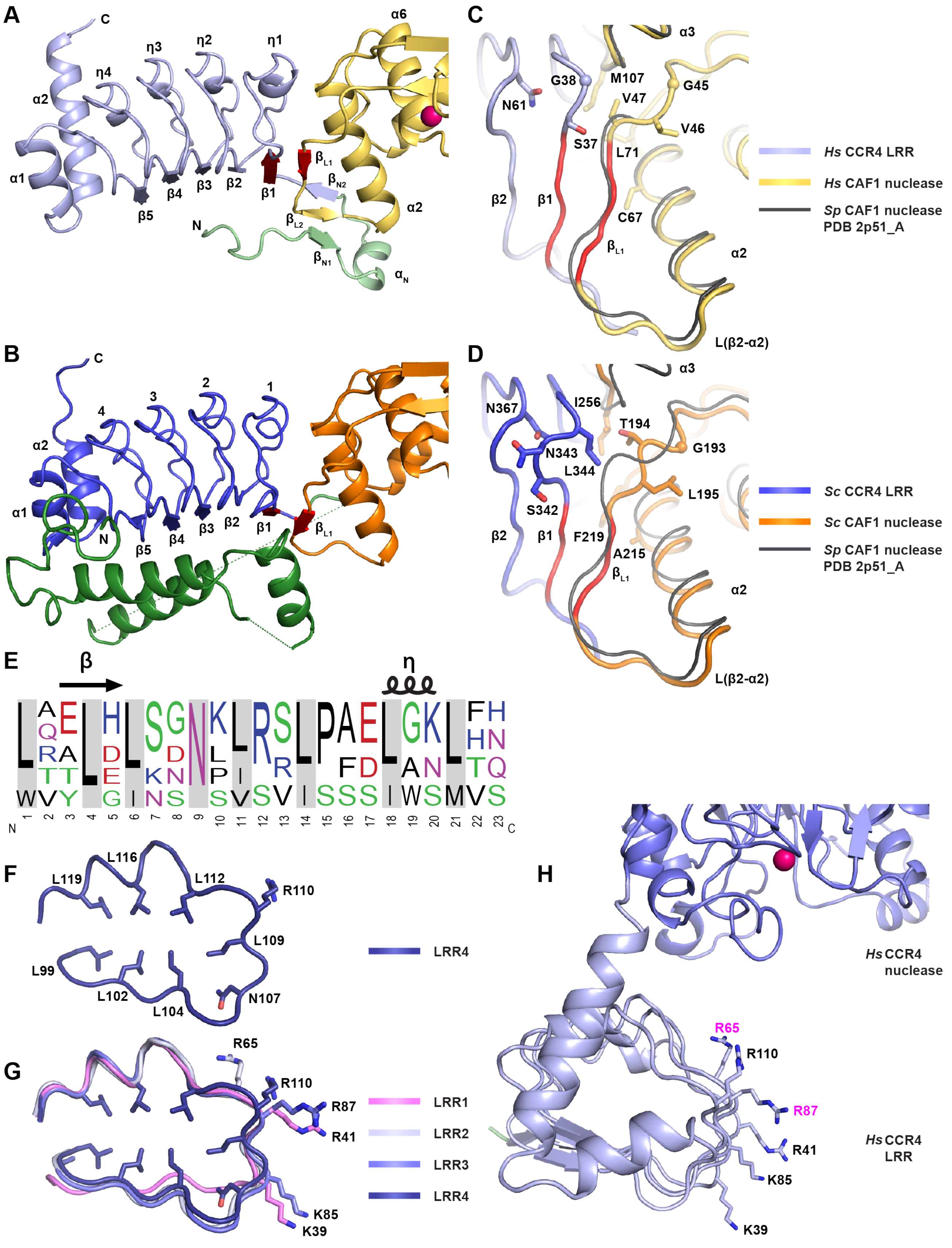

Figure S2. Structural details of CCR4-CAF1 complex formation and of the human CCR4 LRR domain

(A, B) Comparison of interfaces between the CCR4 LRR domain and the CAF1 nuclease in the *Hs* CCR4-CAF1 (A) and *Sc* CCR4-CAF1 (B, PDB-ID 4b8c) (36) complexes. In both complexes, the malleable loop L(β2–α2) of CAF1 adjusts to form an antiparallel β-strand interaction (β_{L1}) with β-strand β1 of the first LRR repeat (red). In the human complex, loop L(β2–α2) furthermore engages in additional β-strand interactions (β_{L2}) with the loop L(β_{N1}–α_N–β_{N2}) of the CCR4 N-terminal extension, resulting in a small and distorted β-sheet (see also Figure S1). The N-terminal extension of *Sc* CCR4 (dark green) does not contact *Sc* CAF1. The N-terminal extensions of *Sc* CCR4 (dark green) and other fungal CCR4 proteins do not align with the N-terminal extension of *Hs* CCR4 (pale green) and other metazoan CCR4 homologs, and CCR4 homologs of plants lack the entire LRR domain (38).

(C, D) Details of the interfaces. Both in the human (C) and in the yeast (D, PDB-ID 4b8c) (36) CCR4-CAF1 complex, hydrophobic residues from CAF1 α-helix α2 and from the loop L(β4–α3) cover the hydrophobic core of the first LRR repeat. In *Hs* CCR4 and its metazoan homologs, a glycine residue (G38) in the first LRR repeat substitutes for the LRR-typical asparagine at this position and for its following residue (N343 and L344 in *Sc* CCR4), causing the repeat to adopt a locally distinct structure that is no longer stabilized by the conserved asparagine from the following LRR repeat (N61 in *Hs* CCR4 LRR2). S37 from *Hs* CCR4 LRR1 contacts V47 from *Hs* CAF1 in the widely conserved 'GVV' sequence (G45-V46-V47), whereas L344 from *Sc* CCR4 would clash with human V47. In *Sc* CAF1, the peptide bonds between the corresponding three residues (G193-T194-L195) are flipped, indicating the 'GVV' sequence to be also adaptable. The superimposed backbone of *Sp* CAF1 (PDB-ID 2p51) (31) is shown in black for comparison, both in panel (C) and in panel (D). The 'GVV' sequence follows after β-strand β2 of CAF1 that hosts the catalytic residues D40 and E42 (see also Figure S1B).

(E) Sequence logo (127) generated from *Hs* CCR4a LRR repeats LRR1–LRR4. The positions of the highly conserved asparagine and of the hydrophobic residues constituting the core of each LRR repeat are shaded (78).

(F) Structure of an LRR repeat (*Hs* CCR4a LRR4). The highly conserved asparagine and the hydrophobic leucine residues constituting the core of the repeat are shown as sticks together with R110, which forms part of the positively charged surface patch of the human CCR4 LRR domain.

(G) Structural superposition of the four human LRR repeats (*Hs* CCR4a LRR1–LRR4). The lack of the conserved arginine in LRR1 (magenta) causes an alternate structure of the repeat at the interface with CAF1. Arginine and lysine residues within the positively charged surface patch of the human CCR4 LRR domain are shown as sticks.

(H) Alternate view of the *Hs* CCR4a LRR domain (see Figure 1F). Arginine and lysine residues within the positively charged surface patch are shown as sticks and labeled in magenta if mutated in this work.

Figure S3. Structural details of the CCR4 and CAF1 nucleases in the human CCR4-CAF1 complex and comparison to RNA substrate recognition in related nucleases

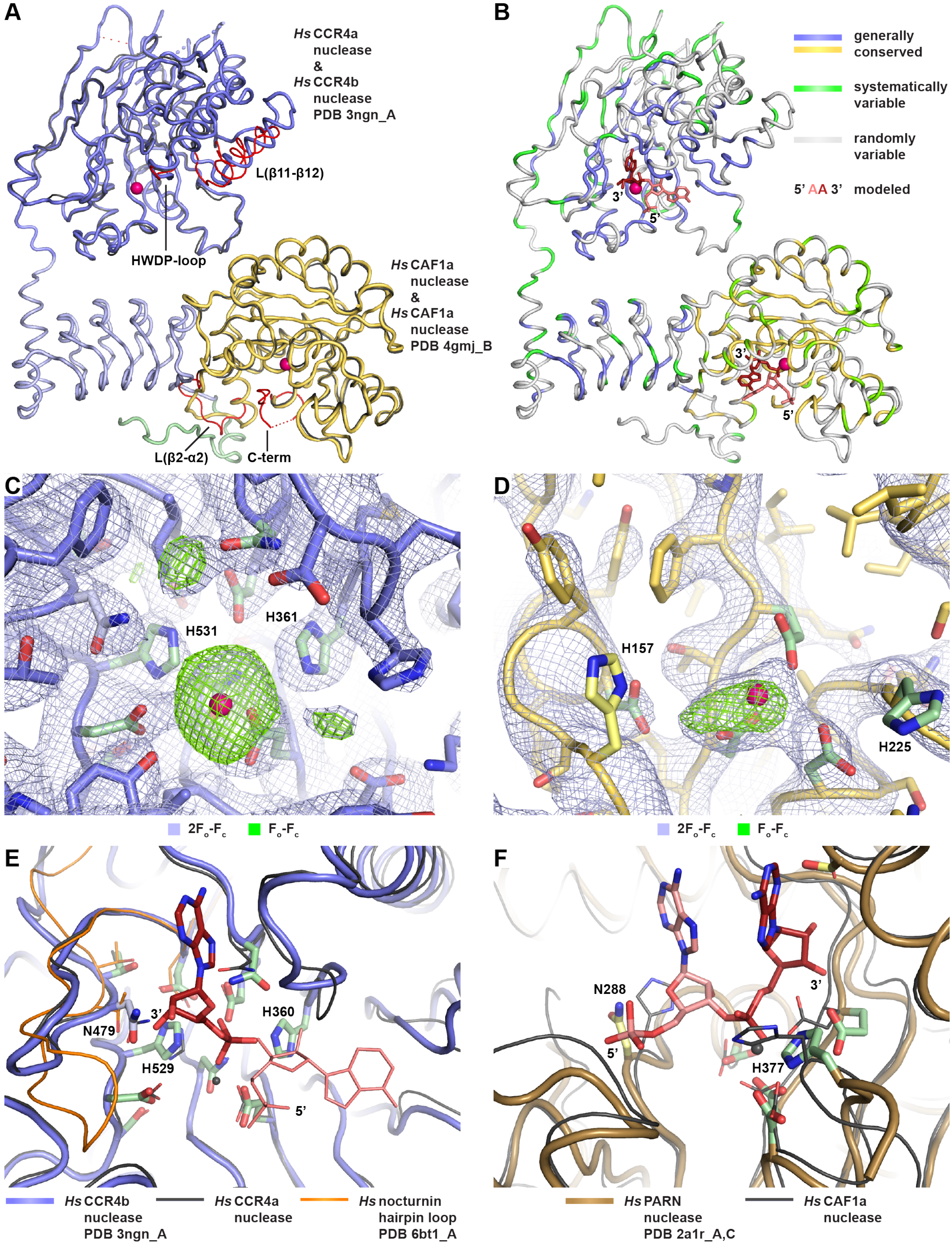

Figure S3. Structural details of the CCR4 and CAF1 nucleases in the human CCR4-CAF1 complex and comparison to RNA substrate recognition in related nucleases

(A) Superposition of alternative CCR4 (29) and CAF1 (48) nuclease structures onto the structure of the *Hs* CCR4-CAF1 complex. The apparently malleable loops in CCR4 and CAF1 and the C-terminal tail of CAF1 are highlighted in red (see also Figure 2).

(B) Sequence conservation mapped onto the structure of the *Hs* CCR4-CAF1 complex. As defined in Figure S1, generally conserved residues are colored in slate or yellow, and residues that vary systematically between CCR4 and CAF1 paralogs are highlighted in green. RNA 3'-terminal nucleotides are shown as sticks and modeled into the nuclease active sites as described in Figure 2.

(C, D) Representative electron density for the active sites of the *Hs* CCR4 nuclease (C) and of the *Hs* CAF1 nuclease (D). The polypeptide backbone is drawn as a tube with side chains shown as sticks. Oxygen atoms are in red, nitrogen atoms are in blue, and the modeled magnesium ions are shown as magenta spheres. Views are similar to Figure 2 with selected residues labeled for orientation. Final electron density (2Fo-Fc type, blue) is contoured at 1.2 sigma. Difference density (Fo-Fc type, green) is calculated in the absence of magnesium ions and contoured at 4.0 sigma. Some of the side chains, such as the histidine residues in the active site of *Hs* CAF1 (H157 and H225), are poorly defined in the electron density, indicating static disorder.

(E) Active site of *Hs* CCR4b crystallized in complex with AMP (PDB-ID 3ngn) (29). The view corresponds to Figure 2. Selected residues (corresponding to *Hs* CCR4a H361 and H531) are labeled for orientation and with residue numbers according to *Hs* CCR4b. The superimposed structure from the *Hs* CCR4a-CAF1a complex is shown in black together with the modeled penultimate nucleotide of the RNA substrate (salmon). The superimposed structure of the hairpin loop from human nocturnin (PDB-ID 6bt1) (96), corresponding to loop L(β18–β19) in *Hs* CCR4a, is shown in orange, including the threonine side chain corresponding to N481 in *Hs* CCR4a (N479 in *Hs* CCR4b).

(F) Active site of human PARN nuclease including the terminal two nucleotides of a co-crystallized 5'-AAA-3' RNA trinucleotide (PDB-ID 2a1r) (20). The view corresponds to Figure 2. Selected residues (corresponding to *Hs* CAF1a H157 and H225) are labeled for orientation and with residue numbers according to PARN. The superimposed structure from the *Hs* CCR4a-CAF1a complex is shown in black.

Figure S4. Quality of CCR4-CAF1 complex preparations used in RNA 3'-deadenylation assays

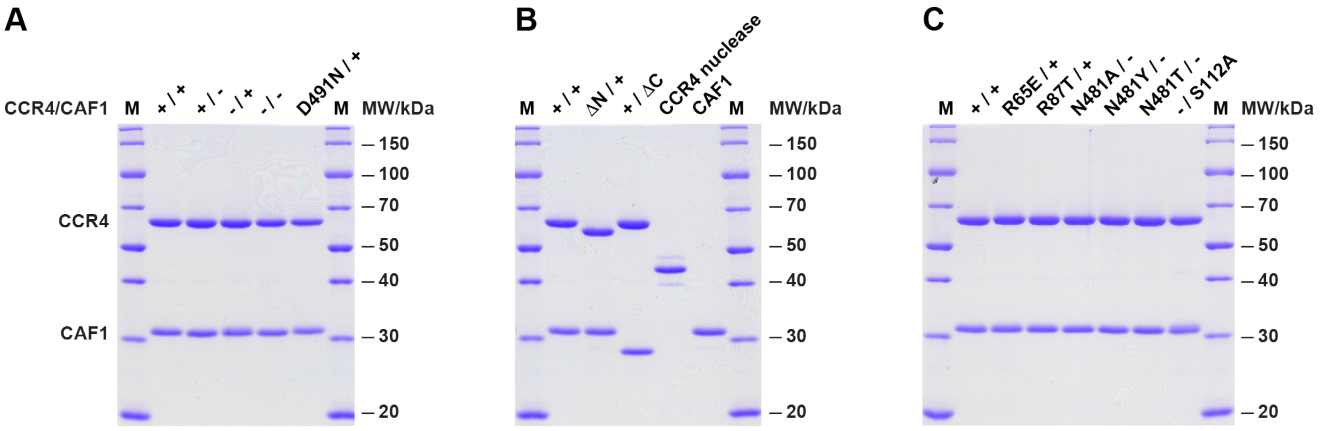

Figure S4. Quality of CCR4-CAF1 complex preparations used in RNA 3'-deadenylation assays

(A-C) Denaturing and Coomassie-stained SDS gels of CCR4-CAF1 complex preparations. Each lane contains 20 pmol of CCR4-CAF1 complex or of the individual CCR4 or CAF1 nucleases, demonstrating protein purity and accuracy of protein quantification in the deadenylation assays. Panels comprise CCR4-CAF1 point mutations of catalytic residues (A), CCR4-CAF1 deletion variants (B) and additional CCR4-CAF1 point mutations (C).

Figure S5. Dependence of RNA 3'-deadenylation on enzyme and RNA substrate concentration and on CCR4-CAF1 complex formation

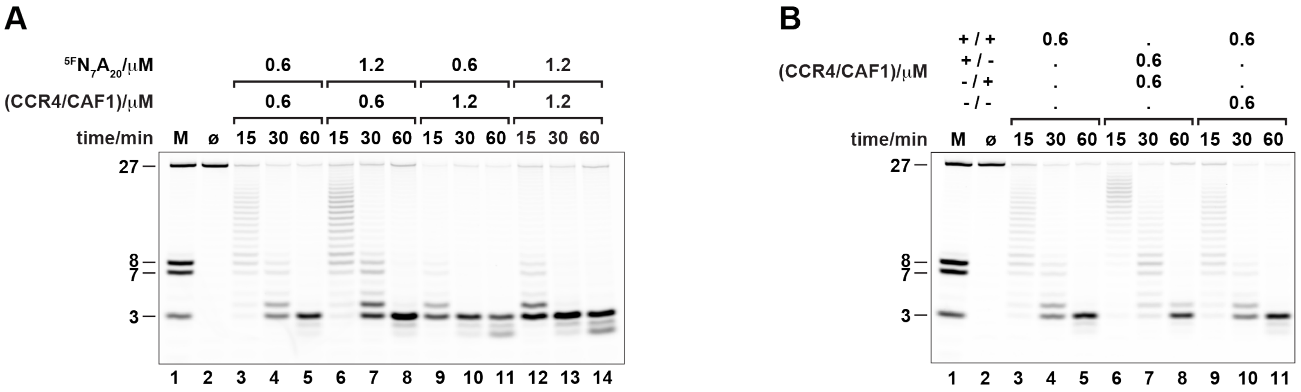

Figure S5. Dependence of RNA 3'-deadenylation on enzyme and RNA substrate concentration and on CCR4-CAF1 complex formation

(A) RNA 3'-deadenylation time course experiments as a function of protein and RNA concentration. Turnover of 5'-FAM-labeled 27-mer RNA substrate ($^{5F}N_7A_{20}$, N_7: UCUAAAU) approximately doubles upon doubling the RNA concentration as indicated by the highly similar deadenylation profiles. Turnover also approximately doubles upon doubling the concentration of the CCR4-CAF1 complex, demonstrating RNA-protein complex formation to be rate-limiting even at micromolar concentrations.

(B) Mixing experiment using active and selectively inactivated versions of the CCR4-CAF1 complex. Protein variants were mixed on ice 10 min before starting the reactions. Deadenylation by CCR4-CAF1 (+/+) is not delayed by the presence of fully inactivated complex (-/-), but a mixture of CCR4 (+/-) and CAF1 (-/+) as parts of selectively inactivated complexes is slightly less efficient, suggesting complex formation to provide a kinetic advantage.

Deadenylation assays were done at 37 °C under standard reaction conditions (50 mM HEPES at pH=7.5, 150 mM NaCl, 2 mM $MgCl_2$ and 1 mM DTT). Reaction products are resolved on denaturing (urea) gels with marker lanes (M) containing RNA oligonucleotides corresponding to the 27 nt substrates or to the most prominent products of RNA hydrolysis with sizes of 8 nt, 7 nt and 3 nt.

Figure S6. Modulation of CCR4 and CAF1 activity by divalent metal ions

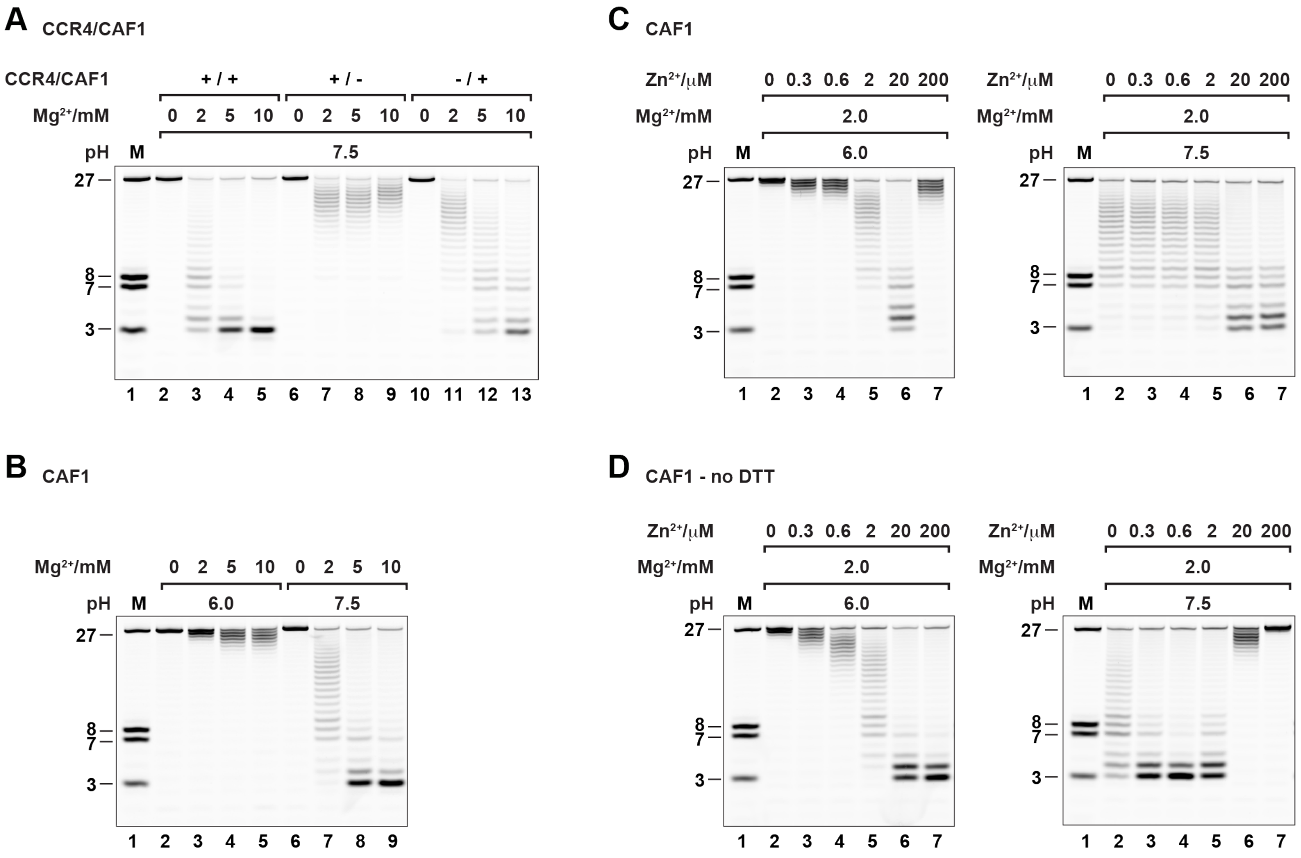

Figure S6. Modulation of CCR4 and CAF1 activity by divalent metal ions

(A, B) Sensitivity of deadenylation by CCR4-CAF1 to the concentration of magnesium ions. Deadenylation reactions were incubated for 30 min at 37 °C under standard reaction conditions with 0.6 μM CCR4-CAF1 complex and 0.6 μM $^{5F}N_7A_{20}$ RNA substrate. In the absence of magnesium ions, both nucleases are inactive. At elevated concentrations of magnesium ions, CCR4 begins to get inhibited, whereas CAF1 gets increasingly stimulated at magnesium ion concentrations as high as 10 mM (A). Stimulation also occurs for the isolated CAF1 nuclease, although at moderately acidic pH the isolated CAF1 nuclease remains only poorly active even at a magnesium ion concentration of 10 mM (B).

(C, D) Stimulation of CAF1-dependent deadenylation by sub-micromolar concentrations of zinc ion. Deadenylation reactions were incubated for 30 min at 37 °C with 0.6 μM CCR4-CAF1 complex and 0.6 μM $^{5F}N_7A_{20}$ RNA substrate and under standard reaction conditions (C, 1 mM DTT) or in the absence of DTT (D). Under standard reaction conditions (C), DTT interferes as a chelator and ligand of zinc ion in a complicated and pH-dependent manner (93,94) that is difficult to decipher. In the absence of DTT (D), zinc ion strongly stimulates CAF1 activity even at pH=6.0 and already at sub-micromolar concentrations. At pH=7.5, the optimal zinc ion concentration (0.6 μM) exactly matches the available CAF1 protein concentration (0.6 μM) in a 1:1 stoichiometry, whereas elevated concentrations above 2.0 μM become strongly inhibitory.

Figure S7. Nucleobase and backbone-specific substrate recognition on both sides of the scissile phosphate

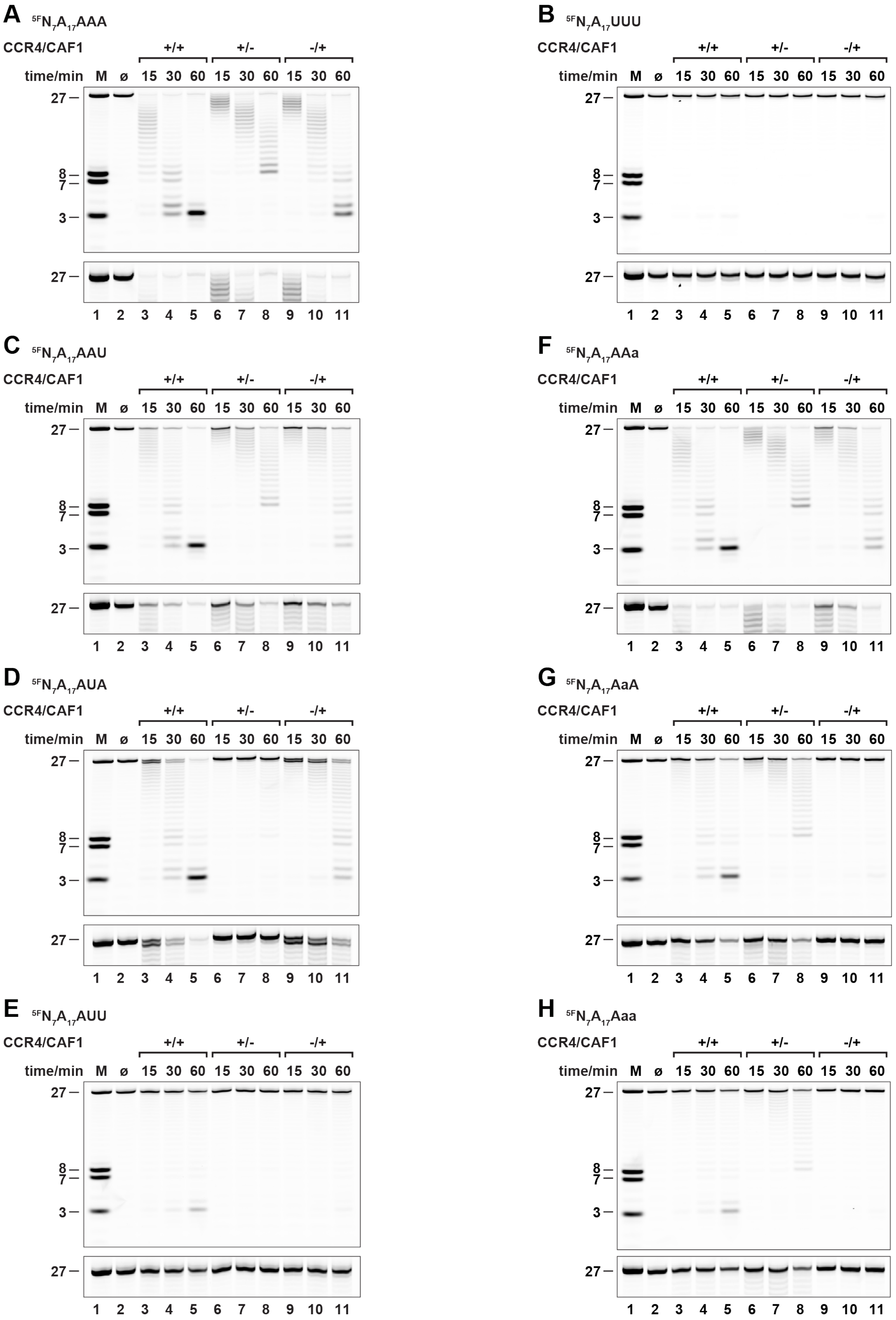

Figure S7. Nucleobase and backbone-specific substrate recognition on both sides of the scissile phosphate

(A-E) Importance of nucleobase identity on both sides of the scissile phosphate. See panel (A) for the unmodified RNA substrate $^{5F}N_7A_{17}AAA$. Uridines were introduced in the last three positions of the oligonucleotide substrate ($^{5F}N_7A_{17}UUU$, B), only in the terminal ($^{5F}N_7A_{17}AAU$, C) or only in the penultimate ($^{5F}N_7A_{17}AUA$, D) positions, or both in the terminal and penultimate positions ($^{5F}N_7A_{17}AUU$, E). In case of the $^{5F}N_7A_{17}AUA$ substrate (D), note the presence of double bands in the 2-fold vertical zoom onto the 27 nt marker region at the bottom of the panel.

(F-H) Importance of the 2'-hydroxyl group on both sides of the scissile phosphate. Deoxyadenines are indicated by lowercase letters and were introduced at the terminal ($^{5F}N_7A_{17}AAa$, F) or penultimate ($^{5F}N_7A_{17}AaA$, G) positions of the oligonucleotide substrate or in both of these positions ($^{5F}N_7A_{17}Aaa$, H). See panel (A) for the unmodified RNA substrate $^{5F}N_7A_{17}AAA$.

Figure S8. Differential discrimination by CCR4 and CAF1 against non-A nucleotides

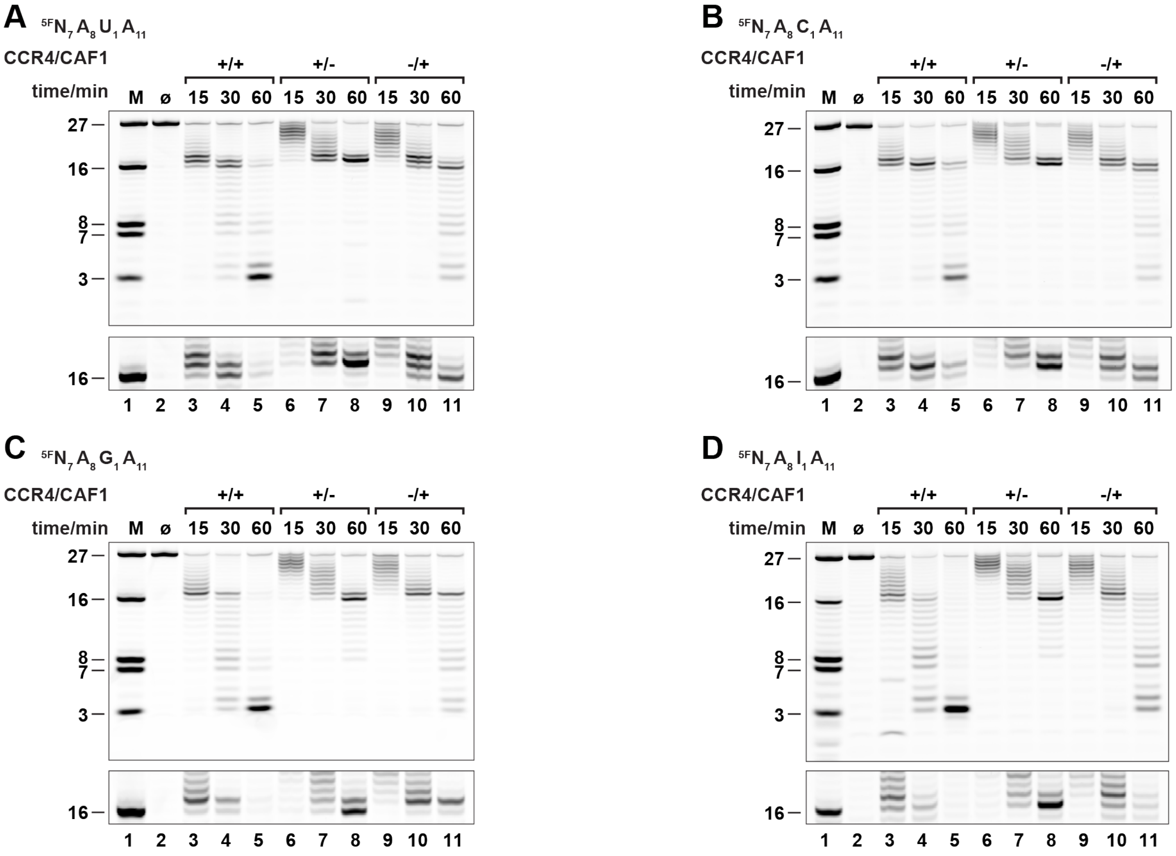

Figure S8. Differential discrimination by CCR4 and CAF1 against non-A nucleotides

(A-D) Discrimination against non-A nucleotides in the active sites of CCR4 (+/-) and CAF1 (-/+). Position A16 of the $^{5F}N_7A_{20}$ RNA substrate was substituted with a pyrimidine (uridine or cytidine, denoted as $^{5F}N_7A_8UA_{11}$ or $^{5F}N_7A_8CA_{11}$) or another purine (guanine or inosine, denoted as $^{5F}N_7A_8GA_{11}$ or $^{5F}N_7A_8IA_{11}$). Note the diversity and the precise position of each of the primary product bands in the 2-fold vertical zoom onto the 16 nt marker region at the bottom of each panel. Importantly and depending on the individual structural context, the non-A nucleotides are frequently rate-limiting already before they reach the 3'-terminal position in the RNA substrate, occasionally even from the pre-penultimate position.

Figure S9. Central structural role of CAF1 in the CCR4-NOT complex and in mRNA deadenylation

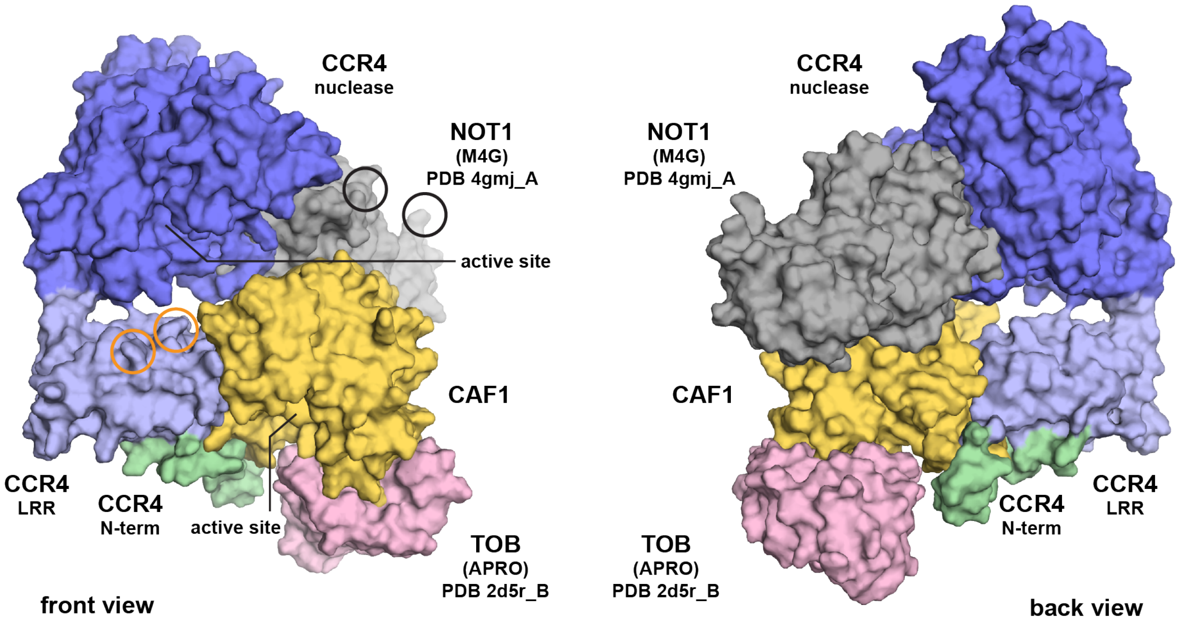

Figure S9. Central structural role of CAF1 in the CCR4-NOT complex and in mRNA deadenylation

The relative orientation of CAF1 partners is illustrated by superimposing the human NOT1 MIF4G domain (PDB-ID 4gmj) and the human TOB APRO domain (PDB-ID 2d5r), as observed in their respective binary complexes with human CAF1 (48,105), onto the structure of the human CCR4-CAF1 complex. This procedure results in only a single and minor clash, involving the NOT1 MIF4G domain and loop L(β9–α7–β10) of the CCR4 nuclease domain (Fig. S1A). The clash could indicate a possible contact in the context of the CCR4-NOT complex, if the flexibly linked CCR4 nuclease slightly adjusts its position. Note that the APRO domain of BTG/TOB proteins does not interfere with the remainder of the assembly and that the active sites, the positively charged patch on the CCR4 LRR domain (orange circles as in Fig. 1G) and the truncated N- and C-termini of the NOT1 MIF4G domain (black circles) do not get occluded. Furthermore, the unknown binding site on CAF1 of the C-terminal region of human SMG7 also remains accessible (116), emphasizing the central role of CAF1 in the assembly of mRNA deadenylation complexes. Left panel: Front view as in Figure 1C, G. Right panel: Back view generated by a rotation of 180° around the y-axis.

Table S1. Plasmid constructs

Plasmid name	Construct details	Backbone*
Hs CCR4a_wt	MBP-HRV3C-*Hs* CCR4a_1–557	pnEA-NpM
Hs CCR4a_mut	MBP-HRV3C-*Hs* CCR4a_1–557 (D491A)	pnEA-NpM
Hs CCR4a_nuc	MBP-HRV3C-*Hs* CCR4a_159–557	pnEA-NpM
Hs CCR4a_R65E	MBP-HRV3C-*Hs* CCR4a_1–557 (R65E)	pnEA-NpM
Hs CCR4a_R87T	MBP-HRV3C-*Hs* CCR4a_1–557 (R87T)	pnEA-NpM
Hs CCR4a_ΔN	MBP-HRV3C-*Hs* CCR4a_29–557	pnEA-NpM
Hs CCR4a_D491N	MBP-HRV3C-*Hs* CCR4a_1–557 (D491N)	pnEA-NpM
Hs CCR4a_N481A	MBP-HRV3C-*Hs* CCR4a_1–557 (N481A)	pnEA-NpM
Hs CCR4a_N481T	MBP-HRV3C-*Hs* CCR4a_1–557 (N481T)	pnEA-NpM
Hs CCR4a_N481Y	MBP-HRV3C-*Hs* CCR4a_1–557 (N481Y)	pnEA-NpM
Hs CAF1a_wt	MBP- HRV3C-*Hs* CAF1a_2–285	pnYC-NpM
Hs CAF1a_mut	MBP- HRV3C-*Hs* CAF1a_2–285 (D40A, E42A)	pnYC-NpM
Hs CAF1a_ΔC	MBP- HRV3C-*Hs* CAF1a_2–263	pnYC-NpM
Hs CAF1a_S112A	MBP- HRV3C-*Hs* CAF1a_2–285 (S112A)	pnYC-NpM

* Plasmid backbones are derived from pET-MCN and described in Diebold *et al*. (2011) (69). Proteolytic removal of the MBP tag leaves an N-terminal 'GPHMLE' sequence preceding each target sequence.

Table S2. CCR4-CAF1 protein complexes

Complex name	CCR4a	CAF1a
CCR4-CAF1 (+/+)	wt	wt
CCR4-CAF1 (+/-)	wt	D40A, E42A
CCR4-CAF1 (-/+)	D491A	wt
CCR4-CAF1 (-/-)	D491A	D40A, E42A
CCR4-CAF1 (R65E/+)	R65E	wt
CCR4-CAF1 (R68T/+)	R68T	wt
CCR4-CAF1 (ΔN/+)	Δ(M1–K28)	wt
CCR4-CAF1 (+/ΔC)	wt	Δ(S264–S285)
CCR4-CAF1 (D491N/+)	D491N	wt
CCR4-CAF1 (N481A/-)	N481A	D40A, E42A
CCR4-CAF1 (N481T/-)	N481T	D40A, E42A
CCR4-CAF1 (N481Y/-)	N481Y	D40A, E42A
CCR4-CAF1 (-/S112A)	D491A	S112A

Table S3. RNA oligonucleotide substrates

Name	Sequence
$^{5F}Y_7A_{20}$	5′-FAM-**UCU** **UCC** **UAA** AAA AAA AAA AAA AAA AAA-3′
$^{5F}N_7A_{20}$<br>$^{5F}N_7A_{17}AAA$	5′-FAM-**UCU** **AAA** **UAA** AAA AAA AAA AAA AAA AAA-3′
$N_7A_{20}{}^{3F}$	5′ **UCU** **AAA** **UAA** AAA AAA AAA AAA AAA AAA-FAM-3′
$^{5F}N_7A_{17}AAA^{PC3}$	5′-FAM-**UCU** **AAA** **UAA** AAA AAA AAA AAA AAA A-A-A-PC3-3′
$^{5F}N_7A_{17}UUU$	5′-FAM-**UCU** **AAA** **UAA** AAA AAA AAA AAA AAA U-U-U-3′
$^{5F}N_7A_{17}AAU$	5′-FAM-**UCU** **AAA** **UAA** AAA AAA AAA AAA AAA A-A-U-3′
$^{5F}N_7A_{17}AUA$	5′-FAM-**UCU** **AAA** **UAA** AAA AAA AAA AAA AAA A-U-A-3′
$^{5F}N_7A_{17}AUU$	5′-FAM-**UCU** **AAA** **UAA** AAA AAA AAA AAA AAA A-U-U-3′
$^{5F}N_7A_{17}AAa$	5′-FAM-**UCU** **AAA** **UAA** AAA AAA AAA AAA AAA rA-rA-dA-3′
$^{5F}N_7A_{17}AaA$	5′-FAM-**UCU** **AAA** **UAA** AAA AAA AAA AAA AAA rA-dA-rA-3′
$^{5F}N_7A_{17}Aaa$	5′-FAM-**UCU** **AAA** **UAA** AAA AAA AAA AAA AAA rA-dA-dA-3′
$^{5F}N_7A_8U_1A_{11}$	5′-FAM-**UCU** **AAA** **UAA** AAA AAA UAA AAA AAA AAA-3′
$^{5F}N_7A_8C_1A_{11}$	5′-FAM-**UCU** **AAA** **UAA** AAA AAA CAA AAA AAA AAA-3′
$^{5F}N_7A_8G_1A_{11}$	5′-FAM-**UCU** **AAA** **UAA** AAA AAA GAA AAA AAA AAA-3′
$^{5F}N_7A_8I_1A_{11}$	5′-FAM-**UCU** **AAA** **UAA** AAA AAA IAA AAA AAA AAA-3′

Supplementary references

20. Wu, M., Reuter, M., Lilie, H., Liu, Y., Wahle, E. and Song, H. (2005) Structural insight into poly(A) binding and catalytic mechanism of human PARN. *EMBO J*, **24**, 4082–4093.

29. Wang, H., Morita, M., Yang, X.N., Suzuki, T., Yang, W., Wang, J.A., Ito, K., Wang, Q.A., Zhao, C., Bartlam, M. *et al.* (2010) Crystal structure of the human CNOT6L nuclease domain reveals strict poly(A) substrate specificity. *EMBO J*, **29**, 2566–2576.

31. Jonstrup, A.T., Andersen, K.R., Van, L.B. and Brodersen, D.E. (2007) The 1.4-A crystal structure of the S. pombe Pop2p deadenylase subunit unveils the configuration of an active enzyme. *Nucleic Acids Res*, **35**, 3153–3164.

36. Basquin, J., Roudko, V.V., Rode, M., Basquin, C., Séraphin, B. and Conti, E. (2012) Architecture of the nuclease module of the yeast Ccr4-not complex: the Not1-Caf1-Ccr4 interaction. *Mol Cell*, **48**, 207–218.

38. Chou, W.L., Chung, Y.L., Fang, J.C. and Lu, C.A. (2017) Novel interaction between CCR4 and CAF1 in rice CCR4-NOT deadenylase complex. *Plant Mol Biol*, **93**, 79–96.

48. Petit, A.P., Wohlbold, L., Bawankar, P., Huntzinger, E., Schmidt, S., Izaurralde, E. and Weichenrieder, O. (2012) The structural basis for the interaction between the CAF1 nuclease and the NOT1 scaffold of the human CCR4-NOT deadenylase complex. *Nucleic Acids Res*, **40**, 11058–11072.

69. Diebold, M.L., Fribourg, S., Koch, M., Metzger, T. and Romier, C. (2011) Deciphering correct strategies for multiprotein complex assembly by co-expression: application to complexes as large as the histone octamer. *J Struct Biol*, **175**, 178–188.

78. Miras, I., Saul, F., Nowakowski, M., Weber, P., Haouz, A., Shepard, W. and Picardeau, M. (2015) Structural characterization of a novel subfamily of leucine-rich repeat proteins from the human pathogen Leptospira interrogans. *Acta Crystallogr D Biol Crystallogr*, **71**, 1351–1359.

93. Krężel, A., Leśniak, W., Jeżowska-Bojczuk, M., Młynarz, P., Brasuñ, J., Kozłowski, H. and Bal, W. (2001) Coordination of heavy metals by dithiothreitol, a commonly used thiol group protectant. *J Inorg Biochem*, **84**, 77–88.

94. Krężel, A. and Maret, W. (2016) The biological inorganic chemistry of zinc ions. *Arch Biochem Biophys*, **611**, 3–19.

96. Abshire, E.T., Chasseur, J., Bohn, J.A., Del Rizzo, P.A., Freddolino, P.L., Goldstrohm, A.C. and Trievel, R.C. (2018) The structure of human Nocturnin reveals a conserved ribonuclease domain that represses target transcript translation and abundance in cells. *Nucleic Acids Res*, **46**, 6257–6270.

105. Horiuchi, M., Takeuchi, K., Noda, N., Muroya, N., Suzuki, T., Nakamura, T., Kawamura-Tsuzuku, J., Takahasi, K., Yamamoto, T. and Inagaki, F. (2009) Structural basis for the antiproliferative activity of the Tob-hCaf1 complex. *J Biol Chem*, **284**, 13244–13255.

116. Loh, B., Jonas, S. and Izaurralde, E. (2013) The SMG5-SMG7 heterodimer directly recruits the CCR4-NOT deadenylase complex to mRNAs containing nonsense codons via interaction with POP2. *Genes Dev*, **27**, 2125–2138.

127. Waterhouse, A.M., Procter, J.B., Martin, D.M., Clamp, M. and Barton, G.J. (2009) Jalview Version 2--a multiple sequence alignment editor and analysis workbench. *Bioinformatics*, **25**, 1189–1191.
